# Bidirectional Perceptual Multimodal Interaction Network Based on Contrastive Learning for Breast Cancer pCR Prediction

**DOI:** 10.3390/tomography12050074

**Published:** 2026-05-19

**Authors:** Jingjing Feng, Zongli Jiang, Jinli Zhang

**Affiliations:** College of Computer Science, Beijing University of Technology, Beijing 100124, China; fengjingjing@emails.bjut.edu.cn (J.F.); jiangzl@bjut.edu.cn (Z.J.)

**Keywords:** breast cancer, pCR prediction, multimodal fusion, contrastive learning

## Abstract

Background/Objectives: Early and accurate prediction of pathological complete response (pCR) after neoadjuvant chemotherapy (NAC) is vital for personalized breast cancer treatment. However, existing deep learning methods are hampered by tumor heterogeneity and semantic misalignment between high-dimensional dynamic contrast-enhanced magnetic resonance imaging (DCE-MRI) and low-dimensional clinical data, which limits pCR prediction performance and generalization. This study addresses these challenges via a novel multimodal network. Methods: We propose a Bidirectional Perceptual Multimodal Interaction Network (BPMINet) based on contrastive learning. BPMINet integrates pre-NAC DCE-MRI and clinical information through three core components: (1) we propose a bidirectional cross-modal attention (BiCMA) fusion mechanism to resolve semantic misalignment and facilitate effective multimodal feature fusion; (2) we design a multimodal contrast-aware feature enhancement (MCFE) module as a key component tightly integrated into the pCR-oriented contrastive learning framework, which serves to boost discriminative power for pCR prediction and improve generalization performance on hard-to-classify samples; (3) we adopt a dual-loss strategy to enable the collaborative optimization of discriminative feature representation and pCR prediction performance. Results: On two publicly available multicenter datasets, BPMINet outperformed all comparative methods across seven evaluation metrics: specifically, it surpassed the top-performing baseline by 5.17% in AUC and 5.24% in accuracy on the MAMA-MIA dataset. More notably, it achieved substantially larger gains of 11.72% in AUC and 7.38% in accuracy on the ISPY1 dataset. Conclusions: BPMINet achieves optimal pCR prediction performance, confirming its superiority and strong generalization ability for multimodal breast cancer pCR prediction.

## Simple Summary

Accurate prediction of chemotherapy response is vital for personalized breast cancer treatment. Existing artificial intelligence technologies often struggle to effectively integrate complex medical imaging with clinical data, restricting the performance of pCR prediction. To tackle this limitation, we propose a multimodal network named BPMINet. The proposed model leverages advanced deep learning techniques to fuse magnetic resonance imaging and clinical information, boosting pCR prediction performance. These findings can assist radiologists in making more precise clinical decisions. In the future, integrating diverse data for long-term monitoring will help establish a more reliable intelligent decision support system for breast cancer treatment.

## 1. Introduction

Breast cancer is the most common malignant tumor in women worldwide, posing a major threat to women’s health due to rising morbidity and mortality. Notably, breast cancer exhibits high heterogeneity, characterized by distinct tumor morphology, specific cellular characteristics, and highly variable treatment responses among different patients [1]. Neoadjuvant chemotherapy (NAC) is pivotal for locally advanced breast cancer or breast conservation candidates, shrinking tumors preoperatively, improving breast conservation rates, and assessing drug sensitivity [2]. Pathological complete response (pCR) is an important indicator of NAC efficacy and is associated with improved prognosis and increased breast-conserving potential, particularly in molecular subtypes such as triple-negative and HER2-positive breast cancer. In luminal subtypes, however, the prognostic value of pCR appears to be less pronounced, as other prognostic factors may also play an important role. Nevertheless, pCR remains a widely used indicator for assessing treatment response in clinical practice. The gold standard for confirming pCR remains the pathological analysis of surgical tissue samples, which is an invasive procedure performed after treatment. This delay can lead to unnecessary chemotherapy toxicity and postpone personalized adjustments for non-pCR patients [3]. Given the low pCR incidence and variable treatment sensitivity, non-invasive preoperative pCR prediction has emerged as a critical research focus. Consequently, this study aims to develop a pCR prediction model integrating pre-NAC dynamic contrast-enhanced magnetic resonance imaging (DCE-MRI) and clinical information to early predict NAC efficacy, thereby facilitating timely personalized treatment and reducing the burden of ineffective chemotherapy for breast cancer patients.

**Figure 1:**
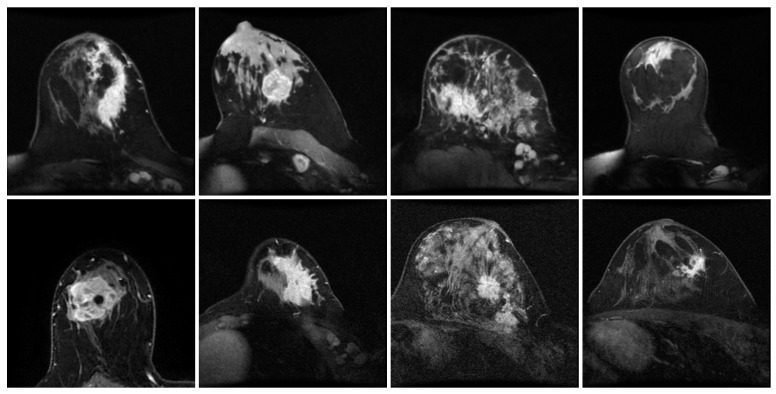
Illustrating the high morphological heterogeneity of breast cancer tumors via DCE-MRI.

DCE-MRI has become an important non-invasive modality for assessing NAC response, offering superior soft-tissue contrast and hemodynamic details compared to traditional X-ray or ultrasound. As shown in Figure 1, DCE-MRI visualizes breast cancer tumors and intuitively reveals their marked morphological heterogeneity, including varied tumor shapes, diverse sizes, and ambiguous lesion boundaries. Such complexity hinders the accurate characterization of lesions for predictive models. While early studies used radiomics-based approaches to predict pCR, these methods relied on artificially designed features and had poor predictive performance and generalization ability [4,5].

Deep learning has advanced rapidly in the fields of natural language processing and computer vision, and is now widely applied in medical research fields including tumor classification, image segmentation, survival analysis, and pCR prediction [6,7]. Multiple studies [8,9,10] have demonstrated that deep learning-based methods achieve superior performance compared to traditional radiomics-based approaches in predicting pCR. Consequently, deep learning has emerged as the mainstream methodology for this task in breast cancer treatment.

Clinical molecular markers, such as estrogen receptor (ER), progesterone receptor (PR), and human epidermal growth factor receptor 2 (HER2), correlate closely with the sensitivity of breast cancer to NAC. Specifically, previous research has demonstrated that ER-negative or low ER-positive patients are more likely to achieve pCR than ER-positive patients [11]. Although DCE-MRI and clinical information can independently predict pCR, multiple studies [12,13,14,15,16] have shown that integrating them through deep learning-based multimodal fusion techniques can leverage their respective strengths and complementary information, thereby improving pCR prediction performance.

However, existing methods for predicting breast cancer pCR using multimodal data still face two primary challenges [17,18,19]. The first challenge is that semantic misalignment between multimodal information limits pCR prediction performance. First, there is significant heterogeneity between high-dimensional DCE-MRI features and clinical information across dimensions, distributions, and granularities. Second, the inherent high heterogeneity of breast cancer and the multifaceted nature of chemotherapy response further complicate the establishment of accurate semantic associations between multimodal fusion representations and pCR status. Most existing methods rely on simple concatenation and shallow fusion to combine imaging and clinical features, failing to establish deep cross-modal semantic correlations. This limitation leads to insufficient mining of complementary information between modalities [13]. Furthermore, while some studies employ unidirectional attention mechanisms, such as clinical-guided imaging feature selection, they fail to capture dynamic bidirectional semantic interactions. This limitation makes it difficult to effectively resolve semantic misalignment, thereby hindering pCR prediction performance [20,21].

The second challenge lies in limited model generalization capability. The distribution of pCR-related features varies considerably among different patients. Coupled with limited clinical sample sizes and class imbalance, this severely restricts the model’s generalization performance [22]. Although traditional contrastive learning (CL) methods have been widely used to improve the generalization ability of learned representations, most existing approaches are primarily designed for unimodal scenarios [23]. When extended to multimodal data, a common strategy is to first concatenate features from different modalities and then apply standard supervised contrastive learning. However, such a straightforward combination often fails to achieve sufficient discriminability. A primary factor is that simple concatenation does not explicitly resolve cross-modal heterogeneity. Imaging and clinical features originate from different representation spaces with distinct statistical properties and semantic meanings. Without proper alignment, the similarity computed in the joint space may be unreliable, making it difficult to establish meaningful relationships between samples. In addition, supervised contrastive learning relies on label-driven alignment, which assumes that samples sharing the same label should be closely clustered in the representation space. However, in pCR prediction, patients with the same label can still exhibit substantial variability in tumor morphology and clinical characteristics. Enforcing overly compact representations under this assumption may obscure meaningful intra-class differences and reduce discriminative capacity. Furthermore, concatenation-based fusion treats all modalities as equally important, without considering that the relevance of imaging and clinical information may vary across patients. This lack of adaptability limits the model’s ability to capture patient-specific patterns. Together, these limitations restrict the effectiveness of directly applying standard contrastive learning to concatenated multimodal features, making it challenging to learn robust and generalizable representations for pCR prediction.

To address these challenges, we propose BPMINet, which comprises three core components designed to handle the complexity of multimodal data. First, the bidirectional cross-modal attention (BiCMA) fusion mechanism resolves feature heterogeneity and semantic misalignment. Specifically, it dynamically calibrates clinical feature weights via imaging-guided clinical attention (IGC-Attention) while focusing on chemotherapy-sensitive imaging regions through clinical-guided imaging attention (CGI-Attention), thereby establishing robust cross-modal semantic links. Second, the multimodal contrast-aware feature enhancement (MCFE) module, tightly integrated into the pCR-oriented contrastive learning framework, aims to enhance the model’s ability to distinguish pCR status accurately. By integrating multimodal perceptual dynamic calibration, clinical semantic-guided feature selection, dual-path feature refinement and contrast-aware dynamic activation, it strengthens the discriminability of multimodal fused features for pCR prediction and improves the generalization capability on hard-to-classify samples. Third, a dual-loss strategy is adopted to facilitate the collaborative optimization of feature representation learning and pCR prediction. This strategy establishes a collaborative training mechanism that unifies discriminative feature refinement with predictive accuracy, thereby enhancing overall pCR prediction performance.

Our contributions are summarized as follows:

- We propose the BiCMA fusion mechanism to address modality heterogeneity and semantic misalignment through dynamic bidirectional calibration, comprising IGC-Attention and CGI-Attention. This mechanism establishes stable cross-modal semantic associations by mining complementary inter-modal information, thereby bridging the gap between high-dimensional imaging features and discrete clinical indicators for pCR prediction.
- We design the MCFE module as a key component tightly integrated into the pCR-oriented contrastive learning framework. Integrating multimodal perceptual dynamic calibration, semantic selection, dual-path refinement, and a feedback mechanism leveraging intra-class similarity for adaptive sample activation, it enhances feature discriminability and generalization by strengthening the activation of hard-to-classify samples and establishes a unique closed-loop interaction between feature refinement and contrastive optimization to ensure robustness to challenging pCR cases.
- Experimental results on two public multicenter breast cancer datasets reveal that BPMINet consistently outperforms existing approaches, confirming the effectiveness of its multimodal interaction mechanism for superior pCR prediction.

## 2. Related Work

Recent advancements in breast cancer pCR prediction have increasingly centered on multimodal fusion and contrastive learning. Despite their preliminary success, the pCR prediction performance of these methods remains constrained by the cross-modal semantic misalignment and the limited adaptability of contrastive learning to complex multimodal fused features.

Shallow multimodal fusion strategies have notable limitations. For instance, Duanmu et al. [13] employed a channel-level multiplication mechanism where clinical features act as global gating weights to modulate MRI feature maps. This method realized basic feature selection but imposed uniform global modulation, treating tumor core, boundary, and background identically while ignoring local spatial heterogeneity. It also adopted a “hard” suppression strategy that discarded valuable imaging features when clinical weights approached zero, a problem exacerbated by noise or data sparsity. Furthermore, the study relied on unidirectional interaction, treating clinical data as a one-way filter for imaging instead of enabling reciprocal refinement of clinical representations. Similarly, Li et al. [14] combined radiomics features and UCTransNet-extracted deep learning features via a disjointed multi-stage pipeline. The study then fused multimodal data through simple vector concatenation and logistic regression, compressing high-dimensional imaging feature maps into static vectors. This process erased tumor spatial heterogeneity and structural semantics, and failed to capture non-linear synergies where clinical indicators dynamically influence imaging interpretation.

Even attention-guided frameworks still underperform. For instance, Li et al. [24] developed a Transformer-based model integrating multi-parameter MRI and RNA-seq data, but it compressed MRI spatial features into one-dimensional vectors via global pooling and adopted unidirectional interaction. The model only aligned genomic data to imaging information, rather than enabling mutual calibration between the two modalities. More recently, CITR-Net [25] incorporated clinicopathologic embeddings into a dual-stream encoder to preserve tumor spatial details. However, this model still used unidirectional clinical guidance without mutual calibration of imaging and clinical semantics, and lacked a dedicated mechanism to refine the discriminability of fused features for hard-to-classify samples. Compounding these technical flaws, most of these methods were validated on small or single-center cohorts. They lack robustness to breast cancer’s inherent heterogeneity, as they cannot optimize fused representations for diverse patient distributions.

For contrastive learning, which enhances feature discriminability by clustering similar samples and separating dissimilar samples in latent space, its application in pCR prediction is poorly tailored to multimodal scenarios. M2Fusion [26] leveraged multi-temporal CL to capture tumor evolution before and after NAC, but it relied on post-treatment imaging data. This reliance renders the model inapplicable for early pre-chemotherapy prediction. The study also treated CL as a temporal alignment tool, rather than a mechanism to refine the pCR classification boundary. RaMA-net [27] used radiomics-guided self-attention to align DCE-MRI and apparent diffusion coefficient (ADC) features, but it depended on hand-crafted radiomics features and focused solely on inter-imaging fusion. The study excluded clinical markers, which are critical for linking macroscopic imaging phenotypes to microscopic pCR status. Across these CL-based models, CL is treated as an auxiliary tool with no customized mechanism to refine the multimodal fused features. This deficiency leads to insufficient pCR discriminability for hard-to-classify samples.

These limitations collectively motivate the development of our BPMINet. Specifically, the proposed BiCMA fusion mechanism enables bidirectional cross-modal calibration to bridge the multimodal semantic gap, while the MCFE module customizes CL for multimodal fused features to improve pCR discriminability and adapt to hard-to-classify samples.

## 3. Materials and Methods

### 3.1. Datasets

We utilized two public multi-center breast cancer datasets, namely ISPY1 and MAMA-MIA, to evaluate the effectiveness of the proposed BPMINet in predicting pCR for breast cancer.

**ISPY1**: The ISPY1 dataset, available via The Cancer Imaging Archive (TCIA) as part of the ACRIN 6657 trial [28], includes patients with T3 breast tumors undergoing NAC, and comprises longitudinal breast DCE-MRI scans designed to assess the efficacy of NAC in patients with stage II or III breast cancer. We used 151 samples selected from a refined version of the ISPY1 dataset [29], where the sample selection was conducted based on the criteria in [30].

**MAMA-MIA**: A large-scale, multi-center benchmark dataset for breast cancer research, comprising 1506 pre-treatment T1-weighted DCE-MRI cases integrated from four TCIA collections [31]. A key strength of MAMA-MIA is its high-quality tumor segmentations verified by 16 clinical experts, coupled with 49 harmonized clinical features that support comprehensive multimodal analysis for pCR prediction. In this study, we excluded 15 cases lacking pCR outcomes, resulting in a final cohort of 1491 cases for training and evaluation.

### 3.2. DCE-MRI Image Preprocessing

The refined version of the ISPY1 dataset underwent a normalization process, which includes bias field correction and resampling to a consistent voxel size of $1\times 1\times 1\;{\mathrm{mm}}^{3}$. For the size normalization of DCE-MRI volumes, we first calculate the number of pixels to be padded in each dimension. Then, we symmetrically pad the volumes with a constant value of 0 along each dimension to expand the volume size. Finally, we crop the central region of the padded volumes to the target size of (192,192,112)(H,W,D). For the MAMA-MIA dataset, we perform standard score (Z-score) normalization and resampling to $1\times 1\times 1\;{\mathrm{mm}}^{3}$ following the protocol in [31], and resize the DCE-MRI volumes to (176,176,160) following the same procedure used for the ISPY1 dataset.

### 3.3. Clinical Information Preprocessing

We use the same seven clinical features on both the ISPY1 and MAMA-MIA datasets [12,13]: age, race, ER status, PR status, hormone receptor (HR) status, HER2 status, and molecular subtype. As shown in Table 1, these features are further categorized into demographic information and clinicopathological information.

In the preprocessing phase, different types of clinical features were handled according to their properties: continuous features, such as age, are standardized using Z-score normalization; binary features, including ER, PR, HR, and HER2 status, retain their original values; and multi-class features, such as race and molecular subtype, are transformed using one-hot encoding.

Missing values were observed in several clinical features within the MAMA-MIA dataset, whereas no missing entries were found in the ISPY1 dataset. As summarized in Table 2, the missing rates for Age, Race, HR, HER2, and Molecular subtype were all below 2%, while ER and PR exhibited substantially higher missing rates of 66.8%.

To maintain a consistent input format, missing entries are handled based on their feature types. Continuous and binary variables including age, ER, PR, HR, and HER2 are assigned a constant value of −1 to denote missingness [32]. For multi-class features, including race and molecular subtype, missing values are assigned as an additional category before one-hot encoding. Specifically, race and molecular subtype are encoded as integer labels from 0 to K−1, where *K* denotes the number of valid categories, and missing values are assigned to the index *K*.

All seven clinical features, including those with missing values, are then concatenated in a fixed order, namely age, race, ER, PR, HR, HER2, and molecular subtype, into a unified clinical information vector and fed into the clinical information encoder described in Section 3.4.2, where feature transformation and normalization are jointly learned. The impact of these variables with high missing rates, specifically ER and PR, is further evaluated through ablation studies in Section 5.3.

### 3.4. Method Overview

**Figure 2:**
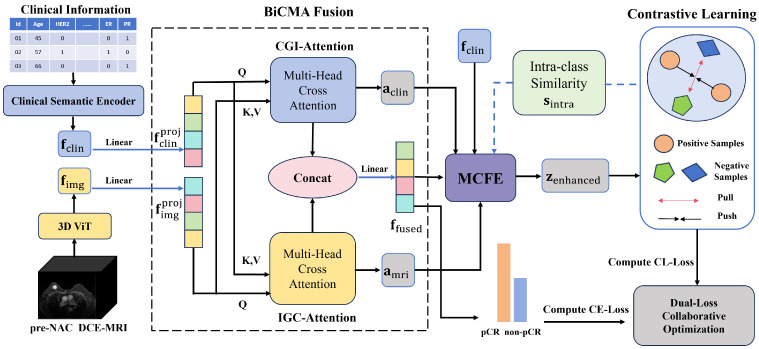
The overall architecture of BPMINet: DCE-MRI images and clinical information are first separately encoded, then fused via the BiCMA fusion mechanism. The multimodal fused feature is enhanced by the MCFE module for the contrastive learning branch, and simultaneously fed into the pCR prediction branch, where the orange bar denotes the pCR class and the blue bar denotes the non-pCR class; during training, both branches are collaboratively optimized by cross-entropy loss for pCR prediction and contrastive learning loss for feature enhancement.

The overall architecture of the BPMINet is shown in Figure 2. BPMINet predicts pCR via four core stages: (1) separate multimodal encoding of DCE-MRI images and clinical information; (2) bidirectional cross-modal attention (BiCMA) fusion to resolve semantic misalignment between multimodal features and generate a multimodal fused feature; (3) multimodal contrast-aware feature enhancement (MCFE) within a contrastive learning framework to enhance pCR discriminability of multimodal fused features; and (4) dual-loss collaborative optimization combining cross-entropy loss (CE-Loss) and contrastive learning loss (CL-Loss) for robust and discriminative pCR prediction.

#### 3.4.1. 3D Vision Transformer for Global Encoding of DCE-MRI

To characterize the complex enhancement patterns and 3D spatial structures of heterogeneous breast tumors [33], we employ a 3D Vision Transformer (ViT) [34] as the feature encoder. By leveraging its global context modeling, the 3D ViT directly captures long-range spatial dependencies within volumetric DCE-MRI data. This approach offers distinct advantages over 2D-based methods in modeling tumor heterogeneity and accommodating the inherent 3D nature of medical imaging. The 3D DCE-MRI volume is denoted as $X\in R^{C\times H\times W\times D}$, where *C* represents the number of channels, (H,W,D) denote spatial dimensions (height, width, depth), *C* value is 1. The feature extraction process of 3D ViT on DCE-MRI volume is as follows:

The 3D DCE-MRI volume *X* is fed into the 3D ViT encoder, and the encoder outputs hidden states of all layers; we extract the hidden state of the last Transformer layer, denoted as $Z^{\mathrm{last}}\in R^{B\times \left(N+1\right)\times C_{\mathrm{hid}}}$, where *B* is the batch size, *N* is the total number of 3D patches, 1 represents the (CLS) token, and $C_{\mathrm{hid}}$ is the hidden dimension. We take the classification (CLS) token, which is the first token in $Z^{\mathrm{last}}$, i.e., $z_{\mathrm{cls}}=Z^{\mathrm{last}}\left[:,0,:\right]\in R^{B\times C_{\mathrm{hid}}}$; this token integrates global 3D features of the DCE-MRI volume. To stabilize the feature distribution and improve subsequent multimodal fusion performance, we add a layer normalization (LayerNorm) operation to the CLS token:

(1) $$f_{\mathrm{img}}=\mathrm{LayerNorm}\left(z_{\mathrm{cls}}\right)$$

where $f_{\mathrm{img}}\in R^{B\times C_{\mathrm{hid}}}$ is the final DCE-MRI feature vector used for subsequent fusion.

#### 3.4.2. Adaptive Clinical Semantic Representation Learning

Given clinical features $x\in R^{d_{\mathrm{clin}}^{\mathrm{in}}}$, where $d_{\mathrm{clin}}^{\mathrm{in}}$ denotes the raw input dimension, this module extracts clinical semantics through a two-stage hierarchical architecture. The process embeds adaptivity into both feature transformation and normalization to capture complex, task-specific clinical relationships.

In the first stage, a linear projection layer with learnable weights $W_{1}\in R^{d_{\mathrm{clin}}^{\mathrm{in}}\times d_{\mathrm{mid}}}$ and biases $b_{1}\in R^{d_{\mathrm{mid}}}$ maps x to an intermediate space $d_{\mathrm{mid}}$, which is set to 128 in our implementation to balance computational efficiency and feature expressiveness. This projection adaptively emphasizes high-impact clinical factors, such as molecular subtypes. Non-linearity is introduced via the Rectified Linear Unit (ReLU) [35], followed by LayerNorm [36] to mitigate inter-individual variability and stabilize the feature distribution:

(2) $$z_{1}=\mathrm{LayerNorm}\left(\mathrm{ReLU}(xW_{1}+b_{1})\right)$$

The second stage further refines the 128-dimensional intermediate features into a 256-dimensional representation via an analogous transformation. Iteratively applying non-linear mapping and distribution stabilization yields the final adaptive clinical semantic representation $f_{\mathrm{clin}}\in R^{256}$, optimized for subsequent cross-modal fusion.

#### 3.4.3. Bidirectional Cross-Modal Attention Fusion Mechanism

The BiCMA fusion mechanism addresses feature heterogeneity and semantic misalignment between DCE-MRI and clinical information, and generates a unified multimodal fused feature. Let $d_{\mathrm{img}}=C_{\mathrm{hid}}=768$ denote the dimension of imaging features $f_{\mathrm{img}}\in R^{d_{\mathrm{img}}}$, and $d_{\mathrm{clin}}=256$ denote the dimension of clinical features $f_{\mathrm{clin}}\in R^{d_{\mathrm{clin}}}$. BiCMA operates in three core stages:

Linear Projection to Shared Space. To address the dimensionality mismatch between the DCE-MRI and clinical information modalities, we first project both features into a shared latent space with a dimension $d_{\mathrm{fuse}}=512$:

(3) $$f_{\mathrm{img}}^{\mathrm{proj}}=W_{\mathrm{img}}f_{\mathrm{img}}+b_{\mathrm{img}},\;f_{\mathrm{clin}}^{\mathrm{proj}}=W_{\mathrm{clin}}f_{\mathrm{clin}}+b_{\mathrm{clin}}$$

where $W_{\mathrm{img}}\in R^{d_{\mathrm{fuse}}\times d_{\mathrm{img}}}$ and $W_{\mathrm{clin}}\in R^{d_{\mathrm{fuse}}\times d_{\mathrm{clin}}}$ are learnable weight matrices, $b_{\mathrm{img}},b_{\mathrm{clin}}\in R^{d_{\mathrm{fuse}}}$ are bias terms, and $f_{\mathrm{img}}^{\mathrm{proj}},f_{\mathrm{clin}}^{\mathrm{proj}}\in R^{d_{\mathrm{fuse}}}$. This transformation maps the heterogeneous features onto a unified dimensional basis, providing a prerequisite for the subsequent bidirectional semantic interaction.

Dual-Stream Cross-Modal Interaction. To achieve comprehensive semantic alignment, BiCMA employs two parallel multi-head cross-attention pathways to construct a bidirectional semantic interaction loop. This design enables mutual refinement and calibration between DCE-MRI semantics and clinical semantics, effectively mitigating cross-modal semantic misalignment. The two core pathways, namely IGC-Attention and CGI-Attention, jointly form the core of this interaction mechanism.

IGC-Attention: Imaging features act as query, with clinical features as keyandvalue to infuse clinical information into imaging semantics. The *H*-head cross attention unfolds as follows:

Multi-Head Projection: Project $f_{\mathrm{img}}^{\mathrm{proj}}$ (query) and $f_{\mathrm{clin}}^{\mathrm{proj}}$ (key/value) into *H* parallel subspaces, where each subspace has a dimension of $d_{\mathrm{head}}=d_{\mathrm{fuse}}/H$:

(4) $$Q_{\mathrm{img}}^{h}=W_{q}^{h}f_{\mathrm{img}}^{\mathrm{proj}},\;K_{\mathrm{clin}}^{h}=W_{k}^{h}f_{\mathrm{clin}}^{\mathrm{proj}},\;V_{\mathrm{clin}}^{h}=W_{v}^{h}f_{\mathrm{clin}}^{\mathrm{proj}}$$

where $W_{q}^{h},W_{k}^{h},W_{v}^{h}\in R^{d_{\mathrm{head}}\times d_{\mathrm{fuse}}}$ are learnable projection matrices for each head h∈{1,2,…,H}. Specifically, $Q_{\mathrm{img}}^{h}\in R^{d_{\mathrm{head}}}$ denotes the Imaging Query that acts as a semantic anchor for cross-modal matching; $K_{\mathrm{clin}}^{h}\in R^{d_{\mathrm{head}}}$ is the Clinical Key used to calculate relevance via similarity with the query; and $V_{\mathrm{clin}}^{h}\in R^{d_{\mathrm{head}}}$ represents the Clinical Value carrying the semantic information to be infused into the imaging features.

Scaled Dot-Product Attention per Head: For each head *h*, we compute attention weights via a scaled dot-product. This product is stabilized by the factor $\sqrt{d_{\mathrm{head}}}$ to prevent gradient explosion. By aggregating clinical value according to these similarity weights, we infuse clinical semantics into the imaging features within the *h*-th subspace:

(5) $${\mathrm{Att}}^{h}=\mathrm{SoftMax}\left(\frac{Q_{\mathrm{img}}^{h}{\left(K_{\mathrm{clin}}^{h}\right)}^{⊤}}{\sqrt{d_{\mathrm{head}}}}\right)V_{\mathrm{clin}}^{h}$$

where ${\mathrm{Att}}^{h}\in R^{d_{\mathrm{head}}}$ denotes the attention output for the *h*-th head, representing imaging features enriched with clinical context specific to this subspace.

Concatenation and Output Projection: We concatenate the outputs from all *H* heads to integrate multi-subspace correlations and project them to the dimension $d_{\mathrm{fuse}}$:

(6) $$f_{\mathrm{img}}^{\mathrm{attn}}=W_{\mathrm{out}}\left[{\mathrm{Att}}^{1}∥{\mathrm{Att}}^{2}∥\ldots ∥{\mathrm{Att}}^{H}\right]$$

where [·∥…∥·] denotes concatenation, and $W_{\mathrm{out}}\in R^{d_{\mathrm{fuse}}\times d_{\mathrm{fuse}}}$ is a learnable matrix. The resulting $f_{\mathrm{img}}^{\mathrm{attn}}\in R^{d_{\mathrm{fuse}}}$ represents the refined imaging feature infused with comprehensive clinical semantics.

CGI-Attention: Clinical features act as query, with imaging features as key and value to infuse imaging features into clinical semantics. The *H*-head cross attention follows the same mechanism:

Multi-Head Projection: Project $f_{\mathrm{clin}}^{\mathrm{proj}}$ (query) and $f_{\mathrm{img}}^{\mathrm{proj}}$ (key/value) into *H* parallel subspaces:

(7) $$Q_{\mathrm{clin}}^{h}=W_{q}^{h}f_{\mathrm{clin}}^{\mathrm{proj}},\;K_{\mathrm{img}}^{h}=W_{k}^{h}f_{\mathrm{img}}^{\mathrm{proj}},\;V_{\mathrm{img}}^{h}=W_{v}^{h}f_{\mathrm{img}}^{\mathrm{proj}}$$

Scaled Dot-Product Attention per Head:

(8) $${\mathrm{Attn}}^{h}=\mathrm{SoftMax}\left(\frac{Q_{\mathrm{clin}}^{h}\;{\left(K_{\mathrm{img}}^{h}\right)}^{⊤}}{\sqrt{d_{\mathrm{head}}}}\right)V_{\mathrm{img}}^{h}$$

Concatenation and Output Projection:

(9) $$f_{\mathrm{clin}}^{\mathrm{attn}}=W_{\mathrm{out}2}\left[{\mathrm{Attn}}^{1}∥{\mathrm{Attn}}^{2}∥\ldots ∥{\mathrm{Attn}}^{H}\right]$$

where $W_{\mathrm{out}2}\in R^{d_{\mathrm{fuse}}\times d_{\mathrm{fuse}}}$ is a learnable matrix. The resulting $f_{\mathrm{clin}}^{\mathrm{attn}}\in R^{d_{\mathrm{fuse}}}$ represents the refined clinical feature, now infused with spatial imaging semantics to resolve the modality-specific misalignment.

Joint Representation Fusion. To synthesize the final discriminative features for classification, the cross-calibrated representations $f_{\mathrm{img}}^{\mathrm{attn}}$ and $f_{\mathrm{clin}}^{\mathrm{attn}}$ are integrated via concatenation and linear projection into a unified 512-dimensional multimodal fused feature $f_{\mathrm{fused}}$:

(10) $$f_{\mathrm{fused}}=W_{\mathrm{fusion}}\left[f_{\mathrm{img}}^{\mathrm{attn}}∥f_{\mathrm{clin}}^{\mathrm{attn}}\right]+b_{\mathrm{fusion}}$$

where $W_{\mathrm{fusion}}\in R^{512\times 2d_{\mathrm{fuse}}}$ and $b_{\mathrm{fusion}}\in R^{512}$ are learnable weight and bias parameters, respectively. By capturing the non-linear correlation between the refined DCE-MRI semantics and clinical indicators, $f_{\mathrm{fused}}$ integrates complementary information from the two modalities. The concatenation followed by linear projection preserves modality-specific characteristics of both attention streams and enables the model to learn adaptive cross-modal interactions through a learnable projection, thereby establishing a unified representation for downstream pCR prediction.

Biological Plausibility of BiCMA. From a clinical perspective, the proposed bidirectional cross-modal attention fusion mechanism can be interpreted as modeling interactions between imaging phenotypes and patient-specific clinical characteristics. In particular, the IGC-Attention pathway allows imaging features to be adaptively re-weighted based on clinical indicators, such as receptor status or molecular subtype, which are known to be associated with tumor heterogeneity and treatment response. Conversely, the CGI-Attention pathway enables clinical representations to capture spatial and morphological cues derived from DCE-MRI, such as tumor enhancement patterns and structural heterogeneity.

This bidirectional interaction is consistent with the clinical decision-making process, where radiological observations are interpreted in the context of patient-specific biological factors, while clinical assessments can also be informed by imaging findings. Therefore, the BiCMA module offers a biologically plausible way to model complementary and interdependent information across modalities, rather than treating imaging and clinical features as independent inputs.

#### 3.4.4. Multimodal Contrast-Aware Feature Enhancement Module

**Figure 3:**
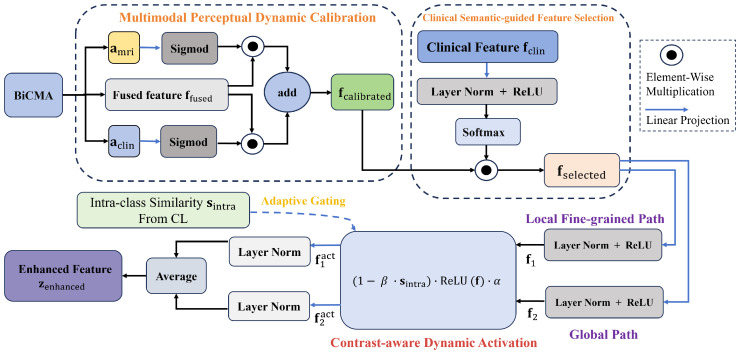
The workflow of the MCFE module is as follows: it takes the multimodal fused feature generated by the BiCMA fusion mechanism along with the cross-modal attention weights as inputs; through multi-layer processing, the module generates the enhanced feature optimized for contrastive learning.

To enhance the discriminative power of the multimodal fused feature $f_{\mathrm{fused}}$, particularly its ability to distinguish between pCR and non-pCR classes and identify hard-to-classify samples, we developed the MCFE module as a key component tightly integrated into the pCR-oriented contrastive learning framework. Its core goal is to highlight key pCR-related information and suppress redundant noise, thereby providing high-quality enhanced features that enable contrastive learning to effectively exploit multimodal data. A workflow of the MCFE processing pipeline is illustrated in Figure 3. The detailed design of MCFE module is as follows:

Multimodal Perceptual Dynamic Calibration. Multimodal fused feature $f_{\mathrm{fused}}$ from BiCMA have achieved semantic alignment, but different samples exhibit varying dependencies on DCE-MRI and clinical features. This mechanism dynamically balances the contribution of two modalities to avoid noise dominance.

Input the cross-modal attention weights from BiCMA, denoted as $a_{\mathrm{mri}}\in R^{B\times 1}$ and $a_{\mathrm{clin}}\in R^{B\times 1}$. Here, $a_{\mathrm{mri}}$ represents the attention intensity of imaging features relative to clinical information, while $a_{\mathrm{clin}}$ signifies the attention intensity of clinical features relative to imaging data. These two weights are derived by aggregating the multi-head cross attention weights of IGC-Attention and CGI-Attention in BiCMA, respectively:

(11) $$a_{\mathrm{mri}}=\frac{1}{H}\sum_{h=1}^{H}\mathrm{GlobalAvgPool}\left(\mathrm{SoftMax}\left(\frac{Q_{\mathrm{img}}^{h}{\left(K_{\mathrm{clin}}^{h}\right)}^{⊤}}{\sqrt{d_{\mathrm{head}}}}\right)\right)$$

(12) $$a_{\mathrm{clin}}=\frac{1}{H}\sum_{h=1}^{H}\mathrm{GlobalAvgPool}\left(\mathrm{SoftMax}\left(\frac{Q_{\mathrm{clin}}^{h}\;{\left(K_{\mathrm{img}}^{h}\right)}^{⊤}}{\sqrt{d_{\mathrm{head}}}}\right)\right)$$

where GlobalAvgPool(·) represents global average pooling over the attention weight matrix, reducing it to a scalar for each sample in the batch.

The sample-specific modal weights $w_{\mathrm{mri}}$ and $w_{\mathrm{clin}}$ are learned via Sigmoid gating to adaptively adjust the modal bias of the multimodal fused feature:

(13) $$\begin{array}{cc} w_{\mathrm{mri}} & =\mathrm{Sigmoid}(W_{\mathrm{mri}}\cdot a_{\mathrm{mri}}+b_{\mathrm{mri}}) \end{array}$$

(14) $$\begin{array}{cc} w_{\mathrm{clin}} & =\mathrm{Sigmoid}(W_{\mathrm{clin}}\cdot a_{\mathrm{clin}}+b_{\mathrm{clin}}) \end{array}$$

where $W_{\mathrm{mri}},W_{\mathrm{clin}}\in R^{1\times 1}$ and $b_{\mathrm{mri}},b_{\mathrm{clin}}\in R^{1}$ are learnable parameters, and $w_{\mathrm{mri}}$ and $w_{\mathrm{clin}}$ quantify the relative contribution of DCE-MRI and clinical features for each individual sample, respectively.

By dynamically measuring modal importance, $w_{\mathrm{mri}}$ and $w_{\mathrm{clin}}$ are utilized to calibrate the multimodal fused feature $f_{\mathrm{fused}}$, yielding the adjusted representation $f_{\mathrm{calibrated}}$:

(15) $$f_{\mathrm{calibrated}}=w_{\mathrm{mri}}⊙f_{\mathrm{fused}}+w_{\mathrm{clin}}⊙f_{\mathrm{fused}}$$

where ⊙ denotes element-wise multiplication, and $w_{\mathrm{mri}}$ and $w_{\mathrm{clin}}$ are automatically broadcast from $R^{B\times 1}$ to match the dimension of $f_{\mathrm{fused}}$ for this operation.

Clinical Semantic-Guided Feature Selection. To focus on pCR-related core features and suppress irrelevant noise, e.g., non-chemosensitivity clinical indicators and background noise in imaging, this mechanism leverages clinical semantic information for feature screening. Generate a feature-wise attention weight s using clinical features $f_{\mathrm{clin}}$ to capture the correlation between clinical semantics and fused features:

(16) $$s=\mathrm{Softmax}\left(\mathrm{ReLU}\left(\mathrm{LayerNorm}(W_{s}\cdot f_{\mathrm{clin}}+b_{s})\right)\right)$$

where $W_{s}\in R^{512\times 256}$ is the projection matrix, $b_{s}\in R^{512}$ is the learnable bias term, and Softmax(·) is employed to normalize the attention weight s.

We obtain the selected feature $f_{\mathrm{selected}}$ by performing element-wise multiplication to highlight information strongly related to pCR:

(17) $$f_{\mathrm{selected}}=f_{\mathrm{calibrated}}⊙s$$

where s represents the attention weight used for feature selection.

Dual-Path Feature Refinement. Two parallel feature processing paths enhance feature expression from local fine-grained information and global information respectively:

Path 1 (Local Fine-Grained Path): This path aims to capture local key details of tumors to enhance local discriminability:

(18) $$f_{1}=\mathrm{ReLU}\left(\mathrm{LayerNorm}(W_{1}\cdot f_{\mathrm{selected}}+b_{1})\right)$$

Path 2 (Global Path): This path focuses on integrating global tumor information to improve noise robustness:

(19) $$f_{2}=\mathrm{ReLU}\left(\mathrm{LayerNorm}(W_{2}\cdot f_{\mathrm{selected}}+b_{2})\right)$$

The two paths share symmetric architectures, $W_{1},W_{2}\in R^{512\times 512}$ and $b_{1},b_{2}\in R^{512}$ denote the learnable weight matrices and bias vectors, respectively. These parameters are optimized to capture diverse feature representations through independent initializations and task-specific training.

Contrast-Aware Dynamic Activation. To better handle hard-to-classify samples in pCR prediction, e.g., borderline cases, this mechanism adaptively adjusts activation intensity based on the intra-class similarity $s_{\mathrm{intra}}$ derived from contrastive learning. This approach enhances the feature distinguishability of challenging samples while preventing overfitting on easy samples.

Specifically, the intra-class similarity $s_{\mathrm{intra}}\in R^{B\times 1}$ is utilized, where smaller values indicate harder samples. It is calculated as the average scaled similarity between sample *i* and its positive samples. The activation intensity is dynamically adjusted to strengthen activations for hard-to-classify samples and weaken them for easy samples:

(20) $$f_{1}^{\mathrm{act}}=\left(1-\beta \cdot s_{\mathrm{intra}}\right)\cdot \mathrm{ReLU}\left(f_{1}\right)\cdot \alpha$$

(21) $$f_{2}^{\mathrm{act}}=\left(1-\beta \cdot s_{\mathrm{intra}}\right)\cdot \mathrm{ReLU}\left(f_{2}\right)\cdot \alpha$$

where α is a global scaling factor and β is a similarity sensitivity factor. Both are learnable parameters that ensure adaptive activation intensity tailored to sample difficulty.

Enhanced Feature Fusion for Contrastive Learning. This step fuses features from the two paths to integrate local fine-grained details with global information, generating the final enhanced feature $z_{\mathrm{enhanced}}$ for subsequent contrastive learning:

(22) $$f_{1}^{\mathrm{proj}}=\mathrm{LayerNorm}\left(W_{1,\mathrm{proj}}\cdot f_{1}^{\mathrm{act}}+b_{1,\mathrm{proj}}\right)$$

(23) $$f_{2}^{\mathrm{proj}}=\mathrm{LayerNorm}\left(W_{2,\mathrm{proj}}\cdot f_{2}^{\mathrm{act}}+b_{2,\mathrm{proj}}\right)$$

Final enhanced feature for contrastive learning is obtained by averaging the projected features above:

(24) $$z_{\mathrm{enhanced}}=\frac{f_{1}^{\mathrm{proj}}+f_{2}^{\mathrm{proj}}}{2}$$

where $z_{\mathrm{enhanced}}\in R^{128}$, $W_{1,\mathrm{proj}},W_{2,\mathrm{proj}}\in R^{128\times 512}$ are learnable matrices, and $b_{1,\mathrm{proj}},b_{2,\mathrm{proj}}$$\in R^{128}$ are learnable bias vectors.

The proposed MCFE module helps preserve intra-class diversity during feature learning. Through multimodal perceptual dynamic calibration and clinical semantic-guided feature selection, the model focuses on pCR-relevant information while suppressing irrelevant variations. In addition, the contrast-aware dynamic activation mechanism adjusts feature representations based on intra-class similarity, allowing samples with heterogeneous characteristics to be refined adaptively rather than being uniformly constrained. This design enables the model to capture shared pCR-related patterns while maintaining meaningful differences among samples, which is particularly important for handling hard-to-distinguish cases in multimodal pCR prediction.

#### 3.4.5. pCR-Oriented Contrastive Learning

To fully leverage the enhanced feature $z_{\mathrm{enhanced}}$ and improve pCR prediction performance under class imbalance, we adopt pCR-oriented contrastive learning [27]. By utilizing $z_{\mathrm{enhanced}}$ for similarity computation and feeding back the resulting intra-class similarity $s_{\mathrm{intra}}$ to the MCFE module, this strategy enables contrast-aware dynamic activation of hard and easy samples. This establishes a unique feedback loop between feature refinement and contrastive learning, a capability absent in conventional contrastive learning approaches.

The key components are defined based on $z_{\mathrm{enhanced}}$ as follows:

- Positive sample set: $P\left(i\right)=\left\{j|y_{j}=y_{i},\;j\neq i\right\}$ comprises all samples in the batch sharing the same pCR label as sample *i*, excluding the sample itself.
- Scaled similarity: $S_{ij}=\frac{z_{\mathrm{enhanced},i}^{⊤}z_{\mathrm{enhanced},j}}{\tau }$, where τ is the temperature parameter used to adjust the similarity distribution.
- Non-self samples: D(i)={k∣k≠i}, representing all samples in the batch except for sample *i* itself.
- Intra-class similarity: $s_{\mathrm{intra},i}=\frac{1}{\left|P(i)\right|}\sum_{j\in P(i)}S_{ij}$, which denotes the average scaled similarity between sample *i* and its positive samples.

The pCR-oriented contrastive learning loss $L_{\mathrm{CL}-\mathrm{pCR}}$ encourages samples with the same pCR label to be closer in the representation space while pushing samples with different labels further apart, thereby enhancing the discriminative power for pCR prediction:

(25) $$L_{\mathrm{CL}-\mathrm{pCR}}=-\frac{1}{N}\sum_{i=1}^{N}\frac{1}{\left|P(i)\right|}\sum_{j\in P(i)}\log\;\left(\frac{\exp(S_{ij})}{\sum_{k\in D(i)}\exp\left(S_{ik}\right)}\right)$$

where *N* represents the total number of samples in the current training batch.

#### 3.4.6. Dual-Loss Collaborative Optimization

For pCR prediction, the multimodal fused feature $f_{\mathrm{fused}}$ is fed into a classification head consisting of a multi-layer perceptron (MLP). To address class imbalance in pCR prediction, this task is trained by minimizing the class-weighted cross-entropy loss $L_{\mathrm{cls}}$, which quantifies the discrepancy between predicted probabilities and ground-truth labels while assigning class-specific weights to pCR and non-pCR classes:

(26) $$L_{\mathrm{cls}}=-\frac{1}{N}\sum_{i=1}^{N}\left(w_{1}\cdot y_{i}\log{\hat{y}}_{i,1}+w_{0}\cdot \left(1-y_{i}\right)\log{\hat{y}}_{i,0}\right)$$

where $y_{i}\in \left\{0,1\right\}$ is the true label of sample *i*, with $y_{i}=1$ denoting pCR and $y_{i}=0$ representing non-pCR; ${\hat{y}}_{i,1}$ is the predicted probability of pCR, and ${\hat{y}}_{i,0}$ is the predicted probability of non-pCR. $w_{1}$ and $w_{0}$ denote the class weights for pCR and non-pCR classes, respectively, and are set to the counts of non-pCR and pCR samples within the training set to mitigate class imbalance.

In order to achieve overall optimization, we adopt a dual-loss collaborative optimization strategy, the total loss combines contrastive learning loss and classification cross-entropy loss to co-optimize discriminative feature representation and pCR prediction performance [37]:

(27) $$L_{\mathrm{total}}=L_{\mathrm{cls}}+\lambda \cdot L_{\mathrm{CL}-\mathrm{pCR}}$$

Hyperparameter sensitivity experiments for contrastive learning demonstrate that the BPMINet achieves optimal performance on both datasets when the temperature parameter τ=0.1 and the contrastive learning loss weight λ=0.9. The comprehensive experimental results and associated discussions are provided in the corresponding section.

## 4. Experiments

### 4.1. Implementation Details

Given that the ISPY1 dataset has no official unified train–test split, most existing methods employed either random fixed-ratio splits or 5-fold cross-validation for model training and evaluation, without publicly releasing details of their specific splits for reference. We thus adopted a 5-fold stratified cross-validation strategy to mitigate random bias in small-sample scenarios [38]. Specifically, the 151 samples were partitioned into a train set consisting of 120 samples and a test set of 31 samples at an 8:2 ratio in each fold, while maintaining the proportion of pCR samples to ensure consistent class distribution across splits.

It is worth noting that, for a fair comparison, the performance results of all comparison methods reported in this study for the ISPY1 dataset were re-run and obtained under the above-mentioned unified dataset split and 5-fold cross-validation strategy consistent with our proposed BPMINet. For the ISPY1 dataset, the performance of our BPMINet and all comparison models is reported as the average of the results derived from the 5-fold cross-validation.

For the MAMA-MIA dataset, we adopted the official pre-defined train–test split to ensure fair comparison with prior studies and consistency with existing benchmarks, although cross-validation could provide a more comprehensive assessment of robustness. Detailed sample distributions for both datasets are summarized in Table 3.

All models in this study were implemented using Python 3.8.19 (https://www.python.org/) and the PyTorch 1.13.0 deep learning framework [39] (https://pytorch.org/), and trained on four servers equipped with NVIDIA GeForce RTX 4090 GPUs (NVIDIA Corporation, Santa Clara, CA, USA). The parameters were configured uniformly for both datasets as follows:

- Optimizer: The AdamW optimizer [40] was employed with a weight decay of 0.01. A layer-wise learning rate strategy was implemented: the initial learning rate for the 3D ViT encoder was set to $10^{-5}$, while the learning rate for other non-pre-trained modules was set to $10^{-4}$. This distinction was made to preserve the pre-trained parameters of the ViT model.
- Learning Rate Scheduler: The CosineAnnealingLR scheduler [41] was utilized to perform periodic adaptive adjustment of the learning rate.
- Hyperparameters: The batch size was set to 4, and the total number of training epochs was set to 50.

### 4.2. Evaluation Metrics

To quantitatively evaluate the comprehensive performance of our BPMINet in pCR prediction, we employed seven widely recognized metrics [30]: Area Under the Curve (AUC), Accuracy (ACC), F1 Score (F1), Positive Predictive Value (PPV), Negative Predictive Value (NPV), Specificity (SPE), and Sensitivity (SEN). In the following tables, bold values indicate the best performance for each metric.

The basic components used for calculation are defined as True Positives (TP), True Negatives (TN), False Positives (FP), and False Negatives (FN). The specific definitions of the seven metrics are as follows:

- Accuracy (ACC): Represents the proportion of correctly classified pCR and non-pCR samples among all cases:
  (28) $$\mathrm{ACC}=\frac{TP+TN}{TP+TN+FP+FN}$$
- Positive Predictive Value (PPV): Also known as Precision, it represents the proportion of samples that the model predicts as pCR and which are actually pCR:
  (29) $$\mathrm{PPV}=\frac{TP}{TP+FP}$$
- Negative Predictive Value (NPV): Evaluates the proportion of samples that the model predicts as non-pCR and which are actually non-pCR:
  (30) $$\mathrm{NPV}=\frac{TN}{TN+FN}$$
- Sensitivity (SEN): Also known as Recall, it quantifies the model’s ability to correctly identify true pCR cases:
  (31) $$\mathrm{SEN}=\frac{TP}{TP+FN}$$
- Specificity (SPE): Quantifies the model’s ability to correctly identify true non-pCR cases:
  (32) $$\mathrm{SPE}=\frac{TN}{TN+FP}$$
- F1 Score (F1): The harmonic mean of PPV and SEN, providing a balanced assessment especially under class imbalance:
  (33) $$F1=\frac{2\times \mathrm{PPV}\times \mathrm{SEN}}{\mathrm{PPV}+\mathrm{SEN}}$$
- Area Under the Curve (AUC): Refers to the area under the Receiver Operating Characteristic (ROC) curve, which plots the True Positive Rate (TPR=SEN) against the False Positive Rate (FPR=1−SPE). Mathematically, AUC quantifies the overall discriminative performance by integrating the TPR over the full range of FPR:
  (34) $$\mathrm{AUC}=\int_{0}^{1}\mathrm{TPR}\left(\mathrm{FPR}\right)\;d\left(\mathrm{FPR}\right)=\int_{0}^{1}\mathrm{SEN}\left(1-\mathrm{SPE}\right)\;d\left(1-\mathrm{SPE}\right)$$
  A higher AUC value signifies a superior ability to distinguish between pCR and non-pCR samples, with a value of 1.0 representing a perfect classifier.

In the primary comparative experiments between BPMINet and competing baseline methods, we further report 95% confidence intervals (CIs) to reflect the uncertainty and robustness of model performance.

For the ISPY1 dataset, where evaluation is conducted using five-fold cross-validation, results are reported as the mean across folds. The associated 95% CIs are computed using Student’s t-distribution to account for the limited number of samples.

On the MAMA-MIA dataset, which follows an official fixed train–test split, performance is evaluated on an independent test set, and the corresponding 95% CIs are estimated via bootstrap resampling with 1000 iterations [42].

In additional experiments, such as ablation studies, validation of clinical information selection, and hyperparameter analysis of contrastive learning, we report mean performance values without confidence intervals, as these experiments are designed to provide controlled comparisons for analyzing the impact of individual components or parameter choices. Including confidence intervals would add complexity without substantially improving interpretability in this context.

## 5. Results

### 5.1. Performance Comparison with Other Methods

To evaluate the performance of BPMINet in predicting pCR to NAC in breast cancer, we compared it with existing excellent methods on the ISPY1 and MAMA-MIA datasets. To quantify the impact of multimodal information on prediction performance, we categorize the comparative methods into the following three groups:

- CI-UM: Unimodal methods leveraging only clinical information.
- DCE-MRI-UM: Unimodal methods leveraging only DCE-MRI images.
- Multimodality: Multimodal methods that leverage multimodal data for predictive tasks.

#### 5.1.1. Unimodal Methods for Clinical Information

We selected two unimodal baseline models relying solely on clinical information: BERT [43], which integrates seven clinical features into a unified text format, and MLP [44], which processes clinical feature embeddings directly. These baselines were selected given the limited development of deep learning methods for pCR prediction based only on clinical data.

The BERT method utilizes a uniform text format. For example, a breast cancer patient has an age of 37.68 and is of Caucasian ethnicity. Before neoadjuvant chemotherapy, the patient’s clinicopathological information is represented as follows: estrogen receptor (ER) status is negative; progesterone receptor (PR) status is negative; hormone receptor (HR) status is positive; human epidermal growth factor receptor 2 (HER2) status is negative; and molecular subtype is triple-negative breast cancer.

The 5-fold cross-validation (5-FCV) results of the BPMINet model on the ISPY1 dataset are presented in Table 4. It can be observed that the performance of 5-FCV exhibits certain fluctuations, the BPMINet achieves optimal performance on Fold 1, attaining an AUC of 0.9545, ACC of 0.9355, F1-score of 0.9, and both NPV and SEN of 1. In contrast, Folds 3 and 4 exhibit relatively lower performance. The final average prediction performance of BPMINet for 5-FCV on the ISPY1 dataset is summarized as follows: AUC = 0.8475, ACC = 0.8452, F1 = 0.7406, PPV = 0.719, NPV = 0.9088, SEN = 0.7778, and SPE = 0.8727.

Due to space constraints, Table 5 and Table 6 report 95% confidence intervals (CIs) only for the four primary metrics (AUC, ACC, SEN, and SPE), while the complete results for all seven metrics are provided in Table A1 and Table A2 in the Appendix A.

As shown in Table 6, BPMINet’s prediction performance on the MAMA-MIA dataset is as follows: AUC = 0.737, ACC = 0.7391, F1 = 0.5618, PPV = 0.5882, NPV = 0.7991, SEN = 0.5376, and SPE = 0.8301. Table 5 and Table 6 demonstrate that BPMINet consistently outperforms CI-UM baselines in overall performance across both datasets. Taking the two most critical metrics as examples, BPMINet achieves an AUC of 0.8475 and an ACC of 0.8452 on ISPY1, substantially exceeding the best baseline of 0.703 and 0.7226. Similarly, on MAMA-MIA, where BPMINet reaches an AUC of 0.737 and an ACC of 0.7391, consistently surpassing the top-performing baselines which attained 0.6513 and 0.6756, respectively. These results highlight the inherent limitations of relying solely on clinical information for pCR prediction.

#### 5.1.2. Unimodal Methods for DCE-MRI Images

We selected two types of unimodal baselines based solely on DCE-MRI images. Classic deep learning models for image classification, including ViT [34], DenseNet [45], ConVit [46], and ResNet-50 [47], all of which were adapted for 3D DCE-MRI volumetric data by replacing standard 2D convolutions with 3D convolutions. Specialized method for breast cancer pCR prediction on DCE-MRI images: SIDLN model [30].

Table 5 and Table 6 demonstrate that BPMINet consistently outperforms the DCE-MRI-UM baselines across both datasets. Specifically, on the ISPY1 dataset, BPMINet achieves higher AUC and ACC scores of 0.8475 and 0.8452, outperforming the best baseline results of 0.6803 and 0.6839. Similarly, on MAMA-MIA, it leads with AUC and ACC scores of 0.737 and 0.7391, surpassing the top-performing baseline values of 0.6688 and 0.6455. This confirms that the unimodal methods using only DCE-MRI images have inherent limitations for pCR prediction.

#### 5.1.3. Multimodal Methods

We evaluated seven multimodal comparison methods, categorized into two groups. First, six representative prediction methods were selected: Interactive-Model [13], TMSS [48], Integrated-Model [49], MRI-RNA [24], CITR-Net [25], and AER-SwinT [32]. Second, we implemented BERT-ViT as a competitive baseline; in this architecture, clinical information and DCE-MRI images are encoded by BERT and 3D ViT, respectively, with their concatenated features fed into an MLP for pCR prediction.

On the ISPY1 dataset (Table 5), BPMINet achieves optimal comprehensive performance over all multimodal comparison methods, with AUC and ACC scores of 0.8475 and 0.8452, respectively, compared to the best baseline AUC of 0.7778 and ACC of 0.7871. Similarly, on MAMA-MIA (Table 6), BPMINet leads all multimodal comparison methods by reaching an AUC of 0.737 and an ACC of 0.7391, surpassing the top baseline results of 0.7008 and 0.7124, respectively.

**Figure 4:**
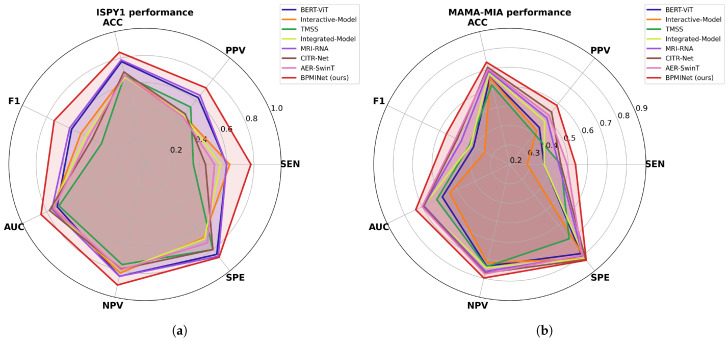
Performance comparison of multimodal methods via radar charts on (**a**) ISPY1 and (**b**) MAMA-MIA datasets.

To intuitively compare the multimodal methods, Figure 4 presents radar charts illustrating performance across all seven metrics. The BPMINet model consistently occupies a larger area than all comparative methods on both datasets, demonstrating optimal comprehensive performance. This multi-metric visualization confirms that the integration of multimodal information in BPMINet is highly effective and robust for breast cancer pCR prediction across different datasets.

#### 5.1.4. Confidence Interval and Statistical Analysis

From the perspective of confidence intervals, BPMINet achieves the best overall performance across all metrics on both datasets. Simultaneously, the widths of its confidence intervals are generally comparable to those of the baseline methods, and are in some cases slightly wider due to the inherent variability of certain metrics such as sensitivity. This suggests that the observed performance improvements offered by BPMINet are not driven by increased variance, but reflect consistent and reliable gains.

It is worth noting that confidence intervals on the ISPY1 dataset were estimated using the t-distribution over cross-validation folds to reflect the variability of model performance. However, this does not constitute a formal statistical significance test between models. In cross-validation settings, the dependence between folds arises from overlapping training data and violates the independence assumptions required by standard paired statistical tests such as DeLong’s test or the bootstrap test [50]. Therefore, formal statistical comparisons are not conducted on ISPY1, while such analysis is performed on the MAMA-MIA dataset due to the availability of an independent test set.

To further assess whether the observed performance improvements on the MAMA-MIA dataset are statistically significant, we conducted formal statistical comparisons between BPMINet and AER-SwinT, which was selected as the best-performing baseline due to its highest AUC on the MAMA-MIA dataset, the primary evaluation metric. It is important to note that the MAMA-MIA test set consists of 306 independent samples. Unlike cross-validation, which averages results across multiple folds, this evaluation was based on a single independent test set and was therefore subject to higher variance.

**Figure 5:**
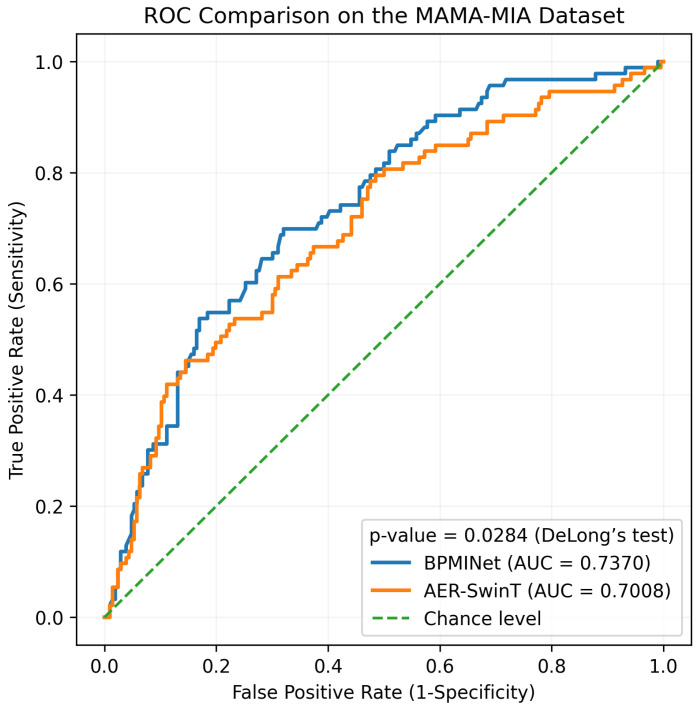
ROC curves of BPMINet and the best-performing baseline AER-SwinT on the MAMA-MIA dataset. BPMINet achieves an AUC of 0.7370, which is higher than the 0.7008 of AER-SwinT. The statistical significance of the AUC improvement is confirmed by DeLong’s test, with a *p*-value = 0.0284.

For a clearer comparison of discriminative performance, the ROC curves of BPMINet and the best-performing baseline AER-SwinT on the MAMA-MIA dataset are shown in Figure 5. BPMINet achieves a higher AUC, and the improvement is statistically significant, as confirmed by DeLong’s test with *p*-value = 0.0284.

Statistical comparisons for the threshold-dependent metrics, including ACC, F1-score, PPV, NPV, sensitivity (SEN), and specificity (SPE), were conducted using paired bootstrap resampling with 1000 iterations. Although statistical significance was not reached at the *p*-value < 0.05 level for metrics such as ACC with *p*-value = 0.0740 and F1-score with *p*-value = 0.0800, all metrics consistently show improved performance, which may be attributed to the moderate sample size and inherent variability in pCR prediction.

### 5.2. Ablation Studies

Overall performance of BiCMA and MCFE-CL. To systematically evaluate the overall performance of the modules within BPMINet, we conducted ablation studies focusing on the proposed BiCMA fusion mechanism and the MCFE module integrated into contrastive learning (MCFE-CL) on two datasets, the quantitative results are presented in Table 7 and Table 8, respectively. In these tables, checkmarks (✓) and crosses (×) denote the inclusion or exclusion of specific modules, respectively. The backbone is common to all variants and is responsible for feature extraction from both DCE-MRI images and clinical data. The base model performs pCR prediction by simple concatenation of these backbone features without BiCMA and MCFE-CL. Model-1 and Model-2 evaluate the independent contributions of BiCMA and MCFE-CL, while our proposed BPMINet integrates all components.

The ablation study results on the ISPY1 and MAMA-MIA datasets consistently demonstrate that the synergistic integration of BiCMA and MCFE-CL is essential for optimal performance. On the ISPY1 dataset, removing both modules in the base model yields the lowest performance with an AUC of 0.6444. In contrast, the inclusion of the BiCMA module alone in Model-1 notably boosts the AUC to 0.7556, marking an improvement of 0.1112. This substantial gain suggests that our cross-modal fusion architecture is inherently effective. When MCFE-CL is introduced individually in Model-2, the AUC reaches 0.7495. Notably, the performance drop observed when removing either BiCMA in Model-2 or MCFE-CL in Model-1 from the full BPMINet is considerable, recorded at 0.098 and 0.0919 respectively. Similar trends are observed on the MAMA-MIA dataset, where the AUC decreases considerably if either the fusion of BiCMA or the contrastive constraint of MCFE-CL is removed.

These findings indicate that the success of BPMINet is not overly reliant on contrastive learning. Instead, BiCMA and MCFE-CL act as complementary components, where BiCMA establishes a robust bidirectional alignment for multimodal features while MCFE-CL serves as a powerful enhancer that refines feature discriminability. Only through their collaborative integration does BPMINet capture both intricate cross-modal correlations and highly discriminative representations, thereby achieving the global optimal performance across all evaluation metrics.

Independent validation of BiCMA mechanism. To further isolate and validate the effectiveness of the proposed BiCMA fusion mechanism and demonstrate the superiority of its bidirectional interaction strategy, we conducted a detailed comparison against alternative multimodal data fusion operations in the scenario without contrastive learning. These alternatives include the concatenation baseline and two unidirectional attention mechanisms: IGC-Attention and CGI-Attention. As shown in Table 9, BiCMA consistently outperforms all unidirectional attention mechanisms and the concatenation baseline on both datasets. For example, on the ISPY1 dataset, BiCMA outperforms CGI-Attention with a substantial increase in AUC and ACC of 44.12% and 33.33%, respectively. Similarly, on the MAMA-MIA dataset, BiCMA yields much higher AUC and ACC scores, substantially surpassing CGI-Attention by 54.57% and 33.76%, respectively. This fully demonstrates the superior ability of our proposed BiCMA module in predicting pCR in breast cancer through its dynamic bidirectional fusion strategy.

Independent validation of MCFE module. To further isolate and validate the internal design effectiveness of the MCFE module, we conducted a component ablation study under the BiCMA-enabled configuration. The comparison results are presented in Table 10. The experimental configurations compare the full MCFE module against two baseline configurations: (i) the version without contrastive learning (W/o CL), and (ii) the conventional contrastive learning framework, which lacks the our proposed MCFE module. As evidenced by Table 10, the BPMINet equipped with the full MCFE yields the best comprehensive performance on both the ISPY1 and MAMA-MIA datasets, surpassing the model without CL and conventional contrastive learning. Crucially, the removal of any core component within the MCFE module, including multimodal dynamic calibration, dual-path refinement, and contrast-aware dynamic activation, leads to a noticeable and consistent performance degradation across all metrics. For example, on the ISPY1 dataset, when MCFE is removed from BPMINet, thereby reducing it to conventional contrastive learning, the AUC drops from 0.8475 to 0.7283, and the SEN decreases from 0.7778 to 0.6889. Similarly, on the MAMA-MIA dataset, the F1-score and SEN of the resulting conventional contrastive learning decrease from 0.5618 to 0.4581 and from 0.5376 to 0.4409, respectively. This strongly validates the necessity and effectiveness of each dedicated component within the MCFE design for enhancing pCR prediction performance.

### 5.3. Validation of Clinical Information Selection

To examine the impact of missing values in our BPMINet model and the adopted preprocessing strategy, we conducted an ablation study on the MAMA-MIA dataset by removing ER and PR, which exhibit relatively high missing rates.

In our clinical information preprocessing pipeline, missing values are handled according to feature types. Continuous and binary variables, including age, ER, PR, HR, and HER2, are assigned a constant value of −1 to indicate missingness. Multi-class variables, including race and molecular subtype, treat missing values as an additional category prior to one-hot encoding. This type-aware strategy ensures a consistent input structure while preserving potentially informative patterns in the data.

As shown in Table 11, the exclusion of ER and PR resulted in a consistent decrease in performance across all evaluation metrics. For example, the AUC decreases from 0.737 to 0.6867, and the sensitivity drops from 0.5376 to 0.4194. These results demonstrate that, despite their high missing rates, ER and PR still contribute valuable discriminative information when available.

Overall, these findings suggest that incorporating incomplete but informative clinical variables can improve the predictive performance of the BPMINet model, highlighting the importance of effectively utilizing partially observed clinical data in multimodal learning.

To further validate the effectiveness and rationale of the comprehensive clinical information set selected for BPMINet [51], we conducted comparative experiments using different clinical feature combinations on two datasets (Table 12). As classified in Table 1, the clinical features are grouped into Clinical 1, comprising demographic data such as age and race, and Clinical 2, which includes clinicopathological factors like ER, PR, HR, HER2, and molecular subtype. Our BPMINet model utilizes the comprehensive set of both Clinical 1 and Clinical 2 features (Clinical 1 & 2).

As shown in Table 12, pCR prediction using clinicopathological factors (Clinical 2) outperforms that of demographic features (Clinical 1). Specifically, Clinical 2 yields substantial increases in AUC and ACC compared to Clinical 1, with improvements of 21.12% and 25.25% on ISPY1 and 36.71% and 13.96% on MAMA-MIA, respectively. This highlights the decisive role of tumor-intrinsic biological factors in treatment response. Nevertheless, the optimal performance is consistently achieved by integrating both sets (Clinical 1 & 2), demonstrating that while clinicopathological markers are primary predictors, demographic features provide essential context. These results validate our strategy of utilizing the full clinical set to capture the multifaceted nature of chemotherapy sensitivity.

### 5.4. Hyperparameter Sensitivity Analysis for Contrastive Learning

The performance of contrastive learning is influenced by the temperature coefficient τ, which regulates the feature similarity distribution, and the CL loss weight λ, which balances the CL loss with the classification loss predicted by pCR. Specifically, a smaller τ produces a sharper similarity distribution, which increases the separation between samples in the feature space and can facilitate the learning of more discriminative representations. The parameter λ controls the trade-off between contrastive representation learning and classification optimization, where different values regulate the relative contribution of the two objectives and influence the balance between feature discrimination and prediction performance.

The candidate ranges τ∈{0.1,0.3,0.5} and λ∈{0.1,0.3,0.5,0.7,0.9,1} were determined by integrating theoretical insights with empirical standards. Specifically, the range for τ covers the values most commonly found to balance class separation and intra-class compactness in classic contrastive learning frameworks such as SimCLR [52] and SupCon [23], where τ=0.1 is a well-established default. For λ, the range was designed to explore a continuum from auxiliary representation learning at λ=0.1 to a balanced joint-optimization at λ=1, ensuring the identification of the optimal balance for the pCR prediction task. To investigate the optimal configuration of contrastive learning for BPMINet, we conducted hyperparameter sensitivity experiments on τ and λ on both the ISPY1 and MAMA-MIA datasets [53,54].

**Figure 6:**
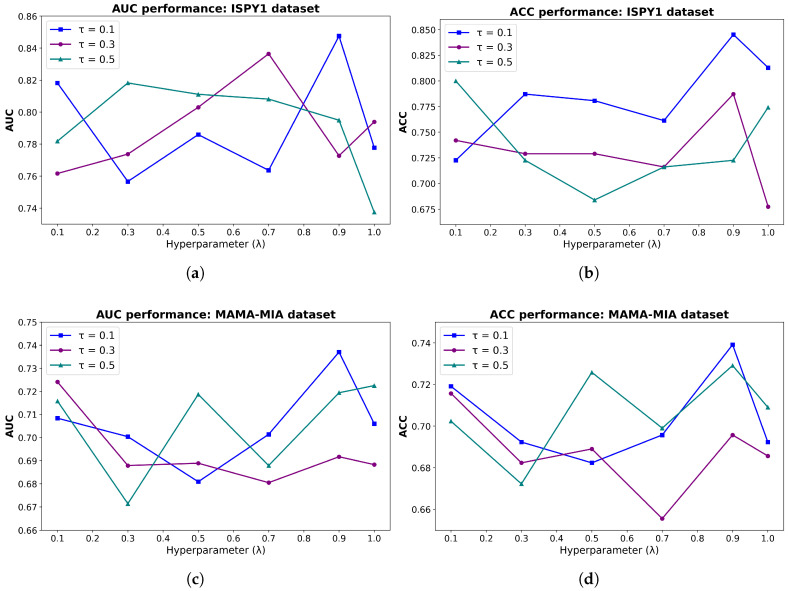
Sensitivity analysis of the contrastive learning module across different λ and τ settings. (**a**) AUC and (**b**) ACC curves on the ISPY1 dataset; (**c**) AUC and (**d**) ACC curves on the MAMA-MIA dataset.

Experimental results summarized in Table 13 and Table 14 demonstrate that the configuration of τ=0.1 and λ=0.9 consistently achieves the global optimal performance across all seven evaluation metrics on both datasets, reflecting strong cross-dataset consistency of the optimal hyperparameters. To visually characterize parameter sensitivity, Figure 6 presents the performance curves using AUC and ACC as representative primary metrics. These curves reveal that BPMINet consistently reaches its efficacy peaks at this specific configuration of τ=0.1 and λ=0.9 on both datasets, manifesting a robust superiority over alternative parameter combinations. Importantly, even in sub-optimal parameter regions, the model maintains a stable performance floor: for instance, most AUC values remain above 0.75 on the ISPY1 dataset, which still outperforms several comparative methods. The observed performance fluctuations demonstrate BPMINet’s sensitivity to τ and λ, but such sensitivity does not compromise the overall stability of the model, thereby justifying our strategic hyperparameter selection and offering empirical guidelines for optimizing contrastive learning in multimodal pCR prediction.

The choice of τ=0.1 aligns with widely adopted settings in classic contrastive learning frameworks, such as SupCon and SimCLR. Our experiments further verify that this value exhibits strong compatibility with the multimodal breast cancer pCR prediction task. Regarding the loss weight λ, while several existing studies [26,55] adopt a default setting of λ=1, our results across two distinct datasets consistently confirm that λ=0.9 is more suitable and yields superior performance for our specific pCR prediction task, further verifying the rationality and generalizability of our hyperparameter selection. To ensure the fairness and consistency of all subsequent experiments involving contrastive learning, we uniformly adopt the experimentally validated optimal hyperparameters for all relevant configurations, where the temperature coefficient τ and the CL loss weight λ are fixed at 0.1 and 0.9, respectively.

### 5.5. Visual Explainability of BPMINet via Grad-CAM

To evaluate the interpretability of BPMINet, we conducted a visual analysis using the Gradient-weighted Class Activation Mapping (Grad-CAM) algorithm [56]. Grad-CAM generates coarse-grained localization maps that highlight regions contributing to the model’s predictions. In these heatmaps, red–yellow hues denote areas with higher activation, while green and blue regions indicate progressively lower contributions. These maps are overlaid on the original DCE-MRI images for intuitive visualization.

**Figure 7:**
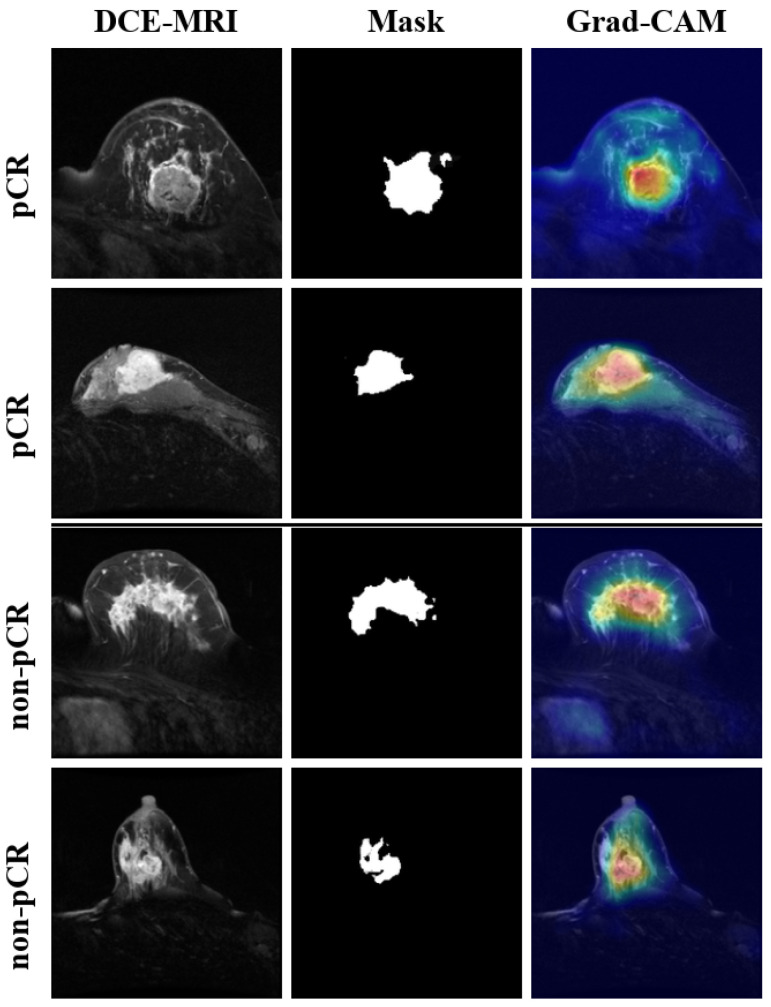
Interpretable visualization: DCE-MRI, Mask, and Grad-CAM for pCR and non-pCR. In the heatmaps, red–yellow regions correspond to higher activation, indicating stronger contributions to the model’s prediction, while green–blue regions denote lower activation.

Figure 7 presents representative examples of DCE-MRI images, tumor segmentation masks, and corresponding Grad-CAM heatmaps for both pCR and non-pCR cases. The highlighted regions generally overlap with tumor areas identified in the segmentation masks, suggesting that the BPMINet model tends to focus on clinically relevant tumor regions rather than background noise when making predictions.

However, it should be noted that Grad-CAM is a post hoc interpretability tool that provides coarse and model-dependent visualizations of feature importance rather than precise localization of pathological biomarkers. The highlighted regions reflect the model’s learned attention patterns and do not necessarily correspond directly to established radiologic markers of treatment response. These visualizations are intended to provide qualitative insights into the model’s decision-making process rather than definitive clinical explanations.

## 6. Discussion

Experimental results show that BPMINet’s comprehensive pCR prediction performance on the ISPY1 and MAMA-MIA datasets is consistently superior to that of all comparative methods (Table 5 and Table 6). Notably, while most existing studies [13,14,24,25,27] rely on relatively small cohorts with fewer than 500 cases, our validation on the large-scale MAMA-MIA dataset comprising 1491 samples provides robust evidence for the generalization capability of BPMINet across heterogeneous multicenter data.

As shown in Table 5 and Table 6, the relative contributions of clinical and imaging modalities vary across datasets. On the ISPY1 dataset, the clinical-only MLP achieves slightly better performance than most imaging-based methods, suggesting that clinical features provide relatively strong predictive signals in this cohort. In contrast, on the MAMA-MIA dataset, imaging-based models such as SIDLN achieve comparable or slightly better performance than clinical-only models. These observations indicate that neither modality consistently dominates across datasets; instead, their effectiveness depends on factors such as sample size, feature completeness, and data distribution.

This variability highlights the importance of integrating complementary information from both modalities. In our model BPMINet, combining clinical and imaging features consistently leads to improved performance, suggesting that the two modalities capture different but mutually informative aspects of treatment response. Ablation experiments further support this observation by demonstrating that the key components of BPMINet, including the BiCMA fusion mechanism and the MCFE module, contribute both individually and jointly to the overall improvement in pCR prediction performance.

A critical challenge in clinical deep learning is model robustness against distribution shift when deployed on out-of-distribution (OOD) data. Our hyperparameter sensitivity analysis for contrastive learning demonstrates that although the contrastive learning component is sensitive to the temperature (τ) and loss weight (λ), the uniform optimal configuration with τ=0.1 and λ=0.9 is consistently obtained across two distinct clinical cohorts, namely ISPY1 and MAMA-MIA. Notably, τ=0.1 is a well-recognized default setting in classic contrastive learning frameworks, and our empirical results further validate its strong suitability for multimodal pCR prediction. Furthermore, while most existing studies conventionally adopt λ=1.0 as a default setting, our two-cohort experiments consistently verify that λ=0.9 achieves better task-adaptive performance.

This cross-dataset consistency suggests that BPMINet has captured generalizable intrinsic patterns for multimodal pCR prediction with cross-cohort generalization capability. Furthermore, the architectural designs of the BiCMA and MCFE modules provide a stable performance baseline, maintaining competitive pCR prediction performance even under sub-optimal hyperparameter settings. Such architectural robustness is crucial to ensuring that the model can be reliably deployed on untuned, completely unknown, out-of-distribution external datasets.

Grad-CAM visualization was employed to verify that BPMINet focuses on core tumor regions rather than non-tumor interference areas. As shown in Figure 7, BPMINet consistently targeted tumor regions, enhancing confidence in its predictive reliability.

To further improve the overall performance of pCR prediction, our future work will focus on three directions: (1) Integrating multi-omics data such as genomics and transcriptomics to complement imaging and clinical features, thus providing a more holistic biological profile for pCR prediction; (2) incorporating medical large language models to infuse the multimodal learning process with rich domain-specific medical prior knowledge, thereby enhancing the clinical interpretability of the model; (3) shifting from static pre-treatment prediction to longitudinal response monitoring by fusing pre-chemotherapy and early-chemotherapy multimodal data, leveraging key dynamic features that more accurately reflect the evolutionary process of pCR.

## 7. Conclusions

We propose BPMINet, a multimodal network that integrates pre-NAC DCE-MRI images and clinical information for breast cancer pCR prediction. Extensive comparative experiments on the ISPY1 and MAMA-MIA datasets demonstrate that BPMINet consistently outperforms all unimodal and multimodal comparison methods. These results validate the efficacy of our fusion approach and its superior generalization capability across diverse patient cohorts. Specifically, BPMINet achieves a superior AUC of 0.8475 and an ACC of 0.8452 on the ISPY1 dataset, while reaching an AUC of 0.737 and an ACC of 0.7391 on the MAMA-MIA dataset. This superior performance is largely attributable to the novel BiCMA fusion mechanism, which resolves semantic misalignment between DCE-MRI and clinical information, and the MCFE module, which enhances feature discriminability of multimodal fused features for pCR prediction. To enhance the interpretability of the prediction process, we utilized the Grad-CAM to generate heatmaps that visualize the focal regions of BPMINet. To further improve the comprehensive performance of breast cancer pCR prediction, our future research will explore integrating medical large language models or multi-omics data, as well as fusing pre-chemotherapy and early-chemotherapy multimodal data to shift from static pre-treatment prediction to longitudinal response monitoring. This aims to establish a more robust intelligent decision-support system for precision breast cancer treatment.

## Figures and Tables

**Table 1:** Classification of clinical information used in the ISPY1 and MAMA-MIA datasets.

Datasets	Demography	Clinicopathology
ISPY1		ER
	Age	PR
	Race	HR
		HER2
		Molecular subtype
MAMA-MIA		ER
	Age	PR
	Race	HR
		HER2
		Molecular subtype

**Table 2:** Summary of missing clinical features in the MAMA-MIA dataset. Percentages are calculated based on the total sample size of 1491.

Feature	Missing (n)	Missing (%)
Age	3	0.20%
Race	16	1.07%
ER	996	66.80%
PR	996	66.80%
HR	16	1.07%
HER2	22	1.48%
Molecular subtype	26	1.74%

**Table 3:** Distribution of pCR samples in the train and test sets used in the two datasets.

Datasets	Categories	pCR	Non-pCR	Total
ISPY1	Train	34	86	120
	Test	9	22	31
				**151** *
MAMA-MIA	Train	347	838	1185
	Test	93	213	306
				**1491** *

* Bold values indicate the total number of samples used in the dataset.

**Table 4:** Results of 5-fold cross validation of BPMINet on the ISPY1 dataset.

Folds	AUC	ACC	SEN	SPE	F1	PPV	NPV
1	0.9545	0.9355	1	0.9091	0.9	0.8182	1
2	0.8838	0.871	0.7778	0.9091	0.7778	0.7778	0.9091
3	0.7172	0.7419	0.4444	0.8636	0.5	0.5714	0.7917
4	0.7677	0.7419	0.7778	0.7273	0.6364	0.5385	0.8889
5	0.9141	0.9355	0.8889	0.9545	0.8889	0.8889	0.9545
Mean ± SD	0.8475 ± 0.0901	0.8452 ± 0.0875	0.7778 ± 0.1859	0.8727 ± 0.0782	0.7406 ± 0.1533	0.719 ± 0.1386	0.9088 ± 0.0699

Note: SD represents the standard deviation.

**Table 5:** Evaluation results of our BPMINet model and all other comparison models on the ISPY1 dataset. For the primary metrics (AUC, ACC, SEN, and SPE), 95% confidence intervals (CIs) are provided in the second line of each cell.

Methods	AUC (95% CI)	ACC (95% CI)	SEN (95% CI)	SPE (95% CI)	F1	PPV	NPV
BERT [43]	0.6909 (0.5842, 0.7936)	0.6968 (0.6124, 0.7831)	0.5555 (0.4218, 0.6842)	0.7546 (0.6632, 0.8415)	0.5147	0.4882	0.8065
MLP [44]	0.7030 (0.6014, 0.8021)	0.7226 (0.6482, 0.7915)	0.4889 (0.3724, 0.6018)	0.8182 (0.7135, 0.9126)	0.5069	0.5504	0.7966
DenseNet [45]	0.6389 (0.5214, 0.7486)	0.6387 (0.5421, 0.7284)	0.4000 (0.2814, 0.5132)	0.7364 (0.6415, 0.8247)	0.3912	0.3873	0.7498
ConVit [46]	0.4889 (0.4021, 0.5732)	0.6709 (0.5846, 0.7512)	0.2222 (0.1142, 0.3325)	0.8545 (0.7712, 0.9324)	0.2826	0.4124	0.7283
ResNet-50 [47]	0.6243 (0.5142, 0.7315)	0.6129 (0.5214, 0.7012)	0.6445 (0.5126, 0.7732)	0.6000 (0.4823, 0.7145)	0.4914	0.4043	0.8065
ViT [34]	0.6495 (0.5512, 0.7431)	0.6839 (0.6012, 0.7642)	0.4889 (0.3621, 0.6084)	0.7636 (0.6642, 0.8561)	0.4762	0.4782	0.7834
SIDLN [30]	0.6803 (0.5912, 0.7645)	0.6774 (0.5842, 0.7621)	0.6222 (0.4614, 0.7752)	0.7000 (0.5842, 0.8124)	0.5161	0.4719	0.8317
BERT-ViT	0.7162 (0.6214, 0.8052)	0.7742 (0.6912, 0.8512)	0.6000 (0.4532, 0.7412)	0.8454 (0.7612, 0.9242)	0.5978	0.6278	0.8430
Interactive-Model [13]	0.7515 (0.6631, 0.8324)	0.6710 (0.5812, 0.7564)	0.6222 (0.5012, 0.7382)	0.6909 (0.5842, 0.7931)	0.5231	0.4588	0.8185
TMSS [48]	0.7010 (0.6123, 0.7842)	0.6710 (0.5814, 0.7562)	0.3556 (0.2124, 0.4952)	0.8000 (0.7012, 0.8941)	0.3541	0.5382	0.7546
Integrated-Model [49]	0.7626 (0.6742, 0.8461)	0.6581 (0.5742, 0.7362)	0.5556 (0.4321, 0.6742)	0.7000 (0.5912, 0.8042)	0.4816	0.4586	0.7992
MRI-RNA [24]	0.7586 (0.6632, 0.8514)	0.7871 (0.7123, 0.8582)	0.6000 (0.4741, 0.7214)	0.8636 (0.7688, 0.9573)	0.6144	0.6491	0.8431
CITR-Net [25]	0.7788 (0.6912, 0.8624)	0.6968 (0.6124, 0.7782)	0.4444 (0.3214, 0.5632)	0.8000 (0.7042, 0.8912)	0.4311	0.4742	0.7882
AER-SwinT [32]	0.7434 (0.6532, 0.8312)	0.6710 (0.5824, 0.7542)	0.5111 (0.3921, 0.6254)	0.7364 (0.6412, 0.8272)	0.4591	0.4484	0.7947
**BPMINet (ours) **	**0.8475** (**0.7356, 0.9594**)	**0.8452** (**0.7366, 0.9538**)	**0.7778** (**0.5470, 0.9989**)	**0.8727** (**0.7756, 0.9698**)	**0.7406**	**0.7190**	**0.9088 **

Note: Bold values indicate the best performance among all compared methods.

**Table 6:** Evaluation results of our BPMINet model and all other comparison models on the MAMA-MIA dataset. For the primary metrics (AUC, ACC, SEN, and SPE), 95% confidence intervals (CIs) are provided in the second line of each cell.

Methods	AUC (95% CI)	ACC (95% CI)	SEN (95% CI)	SPE (95% CI)	F1	PPV	NPV
BERT [43]	0.6503 (0.5986, 0.7022)	0.6321 (0.5816, 0.6826)	0.5054 (0.4274, 0.5832)	0.6893 (0.6324, 0.7388)	0.4608	0.4234	0.7553
MLP [44]	0.6513 (0.5823, 0.7218)	0.6756 (0.6187, 0.7291)	0.3871 (0.2911, 0.4853)	0.8058 (0.7375, 0.8655)	0.4260	0.4737	0.7444
DenseNet [45]	0.5432 (0.4722, 0.6140)	0.6187 (0.5487, 0.6871)	0.2796 (0.1932, 0.3647)	0.7718 (0.7150, 0.8284)	0.3133	0.3562	0.7035
ConVit [46]	0.5451 (0.4748, 0.6163)	0.6321 (0.5821, 0.6853)	0.4194 (0.3401, 0.5035)	0.7282 (0.6649, 0.7872)	0.4149	0.4105	0.7353
ResNet-50 [47]	0.5359 (0.4521, 0.6197)	0.6087 (0.5452, 0.6722)	0.2043 (0.1241, 0.2847)	0.7913 (0.7261, 0.8568)	0.2452	0.3065	0.6878
ViT [34]	0.6053 (0.5392, 0.6724)	0.6455 (0.5591, 0.7322)	0.4086 (0.3027, 0.5143)	0.7524 (0.6632, 0.8452)	0.4176	0.4270	0.7381
SIDLN [30]	0.6688 (0.6050, 0.7295)	0.6421 (0.5741, 0.7114)	0.4946 (0.4251, 0.5622)	0.7087 (0.6444, 0.7650)	0.4623	0.4340	0.7565
BERT-ViT	0.5854 (0.5036, 0.6682)	0.6589 (0.5842, 0.7128)	0.3763 (0.2764, 0.4731)	0.7864 (0.7383, 0.8351)	0.4070	0.4430	0.7364
Interactive-Model [13]	0.5423 (0.4849, 0.6020)	0.6555 (0.5875, 0.7215)	0.2903 (0.2086, 0.3741)	0.8204 (0.7667, 0.8744)	0.3439	0.4219	0.7191
TMSS [48]	0.6170 (0.5406, 0.6845)	0.6187 (0.5617, 0.6724)	0.4624 (0.3636, 0.5647)	0.6893 (0.6321, 0.7391)	0.4300	0.4019	0.7396
Integrated-Model [49]	0.6938 (0.6423, 0.7456)	0.6823 (0.6165, 0.7469)	0.3763 (0.3101, 0.4470)	0.8204 (0.7750, 0.8653)	0.4242	0.4861	0.7445
MRI-RNA [24]	0.6905 (0.6222, 0.7484)	0.6923 (0.6418, 0.7461)	0.4516 (0.3727, 0.5326)	0.8010 (0.7482, 0.8530)	0.4773	0.5060	0.7639
CITR-Net [25]	0.6946 (0.6274, 0.7533)	0.7124 (0.6573, 0.7638)	0.4624 (0.3516, 0.5652)	0.8252 (0.7691, 0.8798)	0.5000	0.5443	0.7727
AER-SwinT [32]	0.7008 (0.6329, 0.7637)	0.7023 (0.6455, 0.7525)	0.4946 (0.3928, 0.5976)	0.7961 (0.7418, 0.8498)	0.5083	0.5227	0.7773
**BPMINet (ours)**	**0.7370** (**0.6730, 0.7953**)	**0.7391** (**0.6854, 0.7862**)	**0.5376** (**0.4338, 0.6324**)	**0.8301** (**0.7812, 0.8814**)	**0.5618**	**0.5882**	**0.7991**

Note: Bold values indicate the best performance among all compared methods.

**Table 7:** Ablation study: overall performance of BiCMA and MCFE-CL on the ISPY1 dataset.

Model	Backbone	BiCMA	MCFE-CL	AUC	ACC	SEN	SPE	F1	PPV	NPV
Base model	✓	×	×	0.6444	0.6516	0.4889	0.7182	0.4524	0.4294	0.7731
Model-1	✓	✓	×	0.7556	0.8	0.6667	0.8545	0.6504	0.6517	0.8671
Model-2	✓	×	✓	0.7495	0.7613	0.6	0.8273	0.5916	0.6077	0.8379
**BPMINet **	✓	✓	✓	**0.8475**	**0.8452 **	**0.7778 **	**0.8727 **	**0.7406 **	**0.719 **	**0.9088 **

Note: Bold values indicate the best performance among all compared methods.

**Table 8:** Ablation study: overall performance of BiCMA and MCFE-CL on the MAMA-MIA dataset.

Model	Backbone	BiCMA	MCFE-CL	AUC	ACC	SEN	SPE	F1	PPV	NPV
Base model	✓	×	×	0.4864	0.6187	0.2796	0.7718	0.3133	0.3562	0.7035
Model-1	✓	✓	×	0.6979	0.689	0.4301	0.8058	0.4624	0.5	0.758
Model-2	✓	×	✓	0.6911	0.6856	0.4409	0.7961	0.4659	0.494	0.7593
**BPMINet **	✓	✓	✓	**0.737 **	**0.7391 **	**0.5376 **	**0.8301 **	**0.5618 **	**0.5882 **	**0.7991 **

Note: Bold values indicate the best performance among all compared methods.

**Table 9:** Performance comparison of BiCMA vs. alternative multimodal data fusion operations in the scenario without contrastive learning.

Datasets	Setting	AUC	ACC	SEN	SPE	F1	PPV	NPV
ISPY1	Concat	0.6444	0.6516	0.4889	0.7182	0.4524	0.4294	0.7731
	IGC-Attention	0.6788	0.6968	0.4667	0.7909	0.47	0.475	0.7844
	CGI-Attention	0.5243	0.6	0.2889	0.7273	0.2841	0.2864	0.7166
	**BiCMA**	**0.7556 **	**0.8 **	**0.6667 **	**0.8545 **	**0.6504 **	**0.6517 **	**0.8671 **
MAMA-MIA	Concat	0.4864	0.6187	0.2796	0.7718	0.3133	0.3562	0.7035
	IGC-Attention	0.6723	0.6388	0.4194	0.7379	0.4194	0.4194	0.7379
	CGI-Attention	0.4515	0.5151	0.2903	0.6165	0.2714	0.2547	0.658
	**BiCMA**	**0.6979 **	**0.689 **	**0.4301 **	**0.8058 **	**0.4624 **	**0.5 **	**0.758 **

Note: Bold values indicate the best performance among all compared methods.

**Table 10:** Performance comparison of MCFE and its ablated variants under the BiCMA-enabled configuration.

Datasets	MCFE Configuration	AUC	ACC	SEN	SPE	F1	PPV	NPV
ISPY1	W/o CL	0.7556	0.8	0.6667	0.8545	0.6504	0.6517	0.8671
	Conventional contrastive learning	0.7283	0.7677	0.6889	0.8	0.6298	0.5874	0.866
	W/o multimodal dynamic calibration	0.7667	0.8129	0.7334	0.8454	0.6912	0.6614	0.8887
	W/o dual-path refinement	0.7677	0.8065	0.6667	0.8636	0.6594	0.6683	0.8674
	W/o contrast-aware dynamic activation	0.8192	0.7161	0.7334	0.7091	0.602	0.5191	0.8677
	**MCFE**	**0.8475 **	**0.8452 **	**0.7778 **	**0.8727 **	**0.7406 **	**0.719 **	**0.9088 **
MAMA-MIA	W/o CL	0.6979	0.689	0.4301	0.8058	0.4624	0.5	0.758
	Conventional contrastive learning	0.6822	0.6756	0.4409	0.7816	0.4581	0.4767	0.7559
	W/o multimodal dynamic calibration	0.691	0.6957	0.4516	0.8058	0.48	0.5122	0.765
	W/o dual-path refinement	0.6867	0.6823	0.4301	0.7961	0.4571	0.4878	0.7558
	W/o contrast-aware dynamic activation	0.6933	0.689	0.4731	0.7864	0.4862	0.5	0.7678
	**MCFE**	**0.737 **	**0.7391 **	**0.5376 **	**0.8301 **	**0.5618 **	**0.5882 **	**0.7991 **

Note: Bold values indicate the best performance among all compared methods.

**Table 11:** Impact of removing ER and PR on the predictive performance of BPMINet on the MAMA-MIA dataset.

Dataset	Setting	AUC	ACC	SEN	SPE	F1	PPV	NPV
MAMA-MIA	w/o ER+PR	0.6867	0.699	0.4194	0.8252	0.4643	0.52	0.7589
	**w/ ER+PR (ours)**	**0.737 **	**0.7391 **	**0.5376 **	**0.8301 **	**0.5618 **	**0.5882 **	**0.7991 **

Note: “w/” and “w/o” denote the inclusion and exclusion of ER and PR features, respectively. Bold values indicate the best performance between the two methods.

**Table 12:** Predictive performance comparison of different clinical information configurations.

Datasets	Setting	AUC	ACC	SEN	SPE	F1	PPV	NPV
ISPY1	Clinical 1	0.6313	0.6387	0.4889	0.7	0.4358	0.413	0.772
	Clinical 2	0.7646	0.8	0.6445	0.8636	0.6453	0.6777	0.8602
	**Clinical 1 & 2 (ours)**	**0.8475 **	**0.8452 **	**0.7778 **	**0.8727 **	**0.7406 **	**0.719 **	**0.9088 **
MAMA-MIA	Clinical 1	0.5168	0.5987	0.3763	0.699	0.3684	0.3608	0.7129
	Clinical 2	0.7065	0.6823	0.5269	0.7524	0.5078	0.49	0.7789
	**Clinical 1 & 2 (ours)**	**0.737 **	**0.7391 **	**0.5376 **	**0.8301 **	**0.5618 **	**0.5882 **	**0.7991 **

Note: Clinical 1 and Clinical 2 represent demographic and clinicopathological information, respectively; “&” denotes the combination of feature sets. Bold values indicate the best performance among all compared methods.

**Table 13:** Impact of hyperparameters λ and τ on the contrastive learning performance within the BPMINet using the ISPY1 dataset.

τ	λ	AUC	ACC	SEN	SPE	F1	PPV	NPV
τ=0.1	0.1	0.8182	0.7226	0.7556	0.7091	0.6102	0.5134	0.8793
	0.3	0.7566	0.7871	0.6222	0.8545	0.6203	0.6433	0.8513
	0.5	0.7859	0.7807	0.6889	0.8182	0.6428	0.6142	0.8682
	0.7	0.7636	0.7613	0.5333	0.8545	0.5426	0.6221	0.8277
	**0.9**	**0.8475 **	**0.8452 **	**0.7778 **	**0.8727 **	**0.7406 **	**0.719 **	**0.9088 **
	1	0.7778	0.8129	0.7334	0.8454	0.6912	0.6614	0.8887
τ=0.3	0.1	0.7616	0.742	0.7333	0.7455	0.6181	0.5364	0.8756
	0.3	0.7737	0.729	0.5556	0.8	0.4852	0.7082	0.8412
	0.5	0.803	0.729	0.6889	0.7455	0.5889	0.527	0.863
	0.7	0.8364	0.7161	0.7111	0.7182	0.5895	0.5248	0.8696
	0.9	0.7727	0.7871	0.6889	0.8273	0.639	0.6858	0.8792
	1	0.7939	0.6774	0.4667	0.7636	0.4024	0.3555	0.7876
τ=0.5	0.1	0.7818	0.8	0.7334	0.8273	0.6817	0.641	0.8838
	0.3	0.8182	0.7226	0.6222	0.7636	0.5641	0.5552	0.8365
	0.5	0.8111	0.6839	0.7334	0.6636	0.5724	0.4758	0.8625
	0.7	0.8081	0.7161	0.6667	0.7364	0.5806	0.5416	0.8462
	0.9	0.7949	0.7226	0.7556	0.7091	0.6164	0.5365	0.8816
	1	0.7374	0.7742	0.7111	0.8	0.6306	0.5828	0.8801

Note: Bold values indicate the best performance among all compared methods.

**Table 14:** Impact of hyperparameters λ and τ on the contrastive learning performance within the BPMINet using the MAMA-MIA dataset.

τ	λ	AUC	ACC	SEN	SPE	F1	PPV	NPV
τ=0.1	0.1	0.7084	0.7191	0.5054	0.8155	0.5281	0.5529	0.785
	0.3	0.7004	0.6923	0.5161	0.7718	0.5106	0.5053	0.7794
	0.5	0.6809	0.6823	0.4624	0.7816	0.4751	0.4886	0.763
	0.7	0.7014	0.6957	0.5161	0.7767	0.5134	0.5106	0.7805
	**0.9**	**0.737 **	**0.7391 **	**0.5376 **	**0.8301 **	**0.5618 **	**0.5882 **	**0.7991 **
	1	0.706	0.6923	0.4194	0.8155	0.4588	0.5065	0.7568
τ=0.3	0.1	0.7241	0.7157	0.4731	0.8252	0.5087	0.55	0.7763
	0.3	0.6879	0.6823	0.4516	0.7864	0.4693	0.4884	0.7606
	0.5	0.6889	0.689	0.4731	0.7864	0.4862	0.5	0.7678
	0.7	0.6805	0.6555	0.5269	0.7136	0.4876	0.4537	0.7696
	0.9	0.6917	0.6957	0.4624	0.801	0.4859	0.5119	0.7674
	1	0.6883	0.6856	0.3871	0.8204	0.4337	0.4932	0.7478
τ=0.5	0.1	0.7158	0.7023	0.4946	0.7961	0.5083	0.5227	0.7773
	0.3	0.6714	0.6722	0.4086	0.7913	0.4368	0.4691	0.7477
	0.5	0.7187	0.7258	0.5269	0.8155	0.5444	0.5632	0.7925
	0.7	0.6879	0.699	0.4516	0.8107	0.4828	0.5185	0.7661
	0.9	0.7194	0.7291	0.5161	0.8252	0.5424	0.5714	0.7907
	1	0.7225	0.709	0.5161	0.7961	0.5246	0.5333	0.7847

Note: Bold values indicate the best performance among all compared methods.

## Data Availability

The data supporting the findings of this study are derived from two publicly accessible datasets: ISPY1 and MAMA-MIA. The ISPY1 dataset is available from The Cancer Imaging Archive (TCIA) at https://www.cancerimagingarchive.net/analysis-result/ispy1-tumor-seg-radiomics/ (accessed on 12 May 2026), while the MAMA-MIA dataset is available on Synapse at https://www.synapse.org/Synapse:syn60868042/wiki/628716 (accessed on 12 May 2026). We release version v1.0 of the code for the 5-fold cross-validation split of the ISPY1 dataset and the BPMINet model on GitHub, at the following URL: https://github.com/peach678/BPMINet (accessed on 12 May 2026).

## Abbreviations

The following abbreviations are used in this manuscript:

pCR	Pathological Complete Response
NAC	Neoadjuvant Chemotherapy
DCE-MRI	Dynamic Contrast-Enhanced Magnetic Resonance Imaging
MRI	Magnetic Resonance Imaging
CNN	Convolutional Neural Network
3D	Three-Dimensional
2D	Two-Dimensional
ADC	Apparent Diffusion Coefficient
TCIA	The Cancer Imaging Archive
TCGA	The Cancer Genome Atlas
Z-score	Standard Score
ER	Estrogen Receptor
PR	Progesterone Receptor
HR	Hormone Receptor
HER2	Human Epidermal Growth Factor Receptor 2
MLP	Multi-Layer Perceptron
LayerNorm	Layer Normalization
ReLU	Rectified Linear Unit
GlobalAvgPool	Global Average Pooling
GPU	Graphics Processing Unit
AdamW	Adam with Weight Decay
CosineAnnealingLR	Cosine Annealing Learning Rate Scheduler
CE	Cross-Entropy
AUC	Area Under the Receiver Operating Curve
ACC	Accuracy
F1	F1 Score
PPV	Positive Predictive Value
NPV	Negative Predictive Value
SPE	Specificity
SEN	Sensitivity
SD	Standard Deviation
5-FCV	5-Fold Cross-Validation
CI	Confidence Interval
BPMINet	Bidirectional Perceptual Multimodal Interactive Network
BiCMA	Bidirectional Cross-Modal Attention
MCFE	Multimodal Contrast-Aware Feature Enhancement
CL	Contrastive Learning
MCFE-CL	MCFE Module Integrated into Contrastive Learning
W/o	Without
Grad-CAM	Gradient-Weighted Class Activation Mapping
IGC-Attention	Imaging-Guided Clinical Attention
CGI-Attention	Clinical-Guided Imaging Attention

## Appendix A

For completeness, this appendix provides detailed experimental data in addition to the main results discussed in the main text. Table A1 and Table A2 list the 95% confidence intervals for all seven evaluation metrics on the ISPY1 and MAMA-MIA datasets, respectively, covering BPMINet and all other comparative methods.

**Table A1:** Evaluation results of our BPMINet model and all other comparison models on the ISPY1 dataset. For all metrics, 95% confidence intervals (CIs) are provided in the second line of each cell.

Methods	AUC (95% CI)	ACC (95% CI)	SEN (95% CI)	SPE (95% CI)	F1 (95% CI)	PPV (95% CI)	NPV (95% CI)
BERT [43]	0.6909 (0.5842, 0.7936)	0.6968 (0.6124, 0.7831)	0.5555 (0.4218, 0.6842)	0.7546 (0.6632, 0.8415)	0.5147 (0.4023, 0.6214)	0.4882 (0.3741, 0.6053)	0.8065 (0.7328, 0.8742)
MLP [44]	0.7030 (0.6014, 0.8021)	0.7226 (0.6482, 0.7915)	0.4889 (0.3724, 0.6018)	0.8182 (0.7135, 0.9126)	0.5069 (0.4125, 0.5982)	0.5504 (0.4231, 0.6742)	0.7966 (0.7314, 0.8583)
DenseNet [45]	0.6389 (0.5214, 0.7486)	0.6387 (0.5421, 0.7284)	0.4000 (0.2814, 0.5132)	0.7364 (0.6415, 0.8247)	0.3912 (0.2831, 0.4952)	0.3873 (0.2741, 0.4936)	0.7498 (0.6712, 0.8235)
ConVit [46]	0.4889 (0.4021, 0.5732)	0.6709 (0.5846, 0.7512)	0.2222 (0.1142, 0.3325)	0.8545 (0.7712, 0.9324)	0.2826 (0.1654, 0.3981)	0.4124 (0.2641, 0.5582)	0.7283 (0.6652, 0.7891)
ResNet-50 [47]	0.6243 (0.5142, 0.7315)	0.6129 (0.5214, 0.7012)	0.6445 (0.5126, 0.7732)	0.6000 (0.4823, 0.7145)	0.4914 (0.3952, 0.5831)	0.4043 (0.3124, 0.4952)	0.8065 (0.7341, 0.8732)
ViT [34]	0.6495 (0.5512, 0.7431)	0.6839 (0.6012, 0.7642)	0.4889 (0.3621, 0.6084)	0.7636 (0.6642, 0.8561)	0.4762 (0.3721, 0.5742)	0.4782 (0.3614, 0.5892)	0.7834 (0.7126, 0.8514)
SIDLN [30]	0.6803 (0.5912, 0.7645)	0.6774 (0.5842, 0.7621)	0.6222 (0.4614, 0.7752)	0.7000 (0.5842, 0.8124)	0.5161 (0.4124, 0.6152)	0.4719 (0.3712, 0.5684)	0.8317 (0.7512, 0.9042)
BERT-ViT	0.7162 (0.6214, 0.8052)	0.7742 (0.6912, 0.8512)	0.6000 (0.4532, 0.7412)	0.8454 (0.7612, 0.9242)	0.5978 (0.4831, 0.7052)	0.6278 (0.5124, 0.7381)	0.8430 (0.7712, 0.9124)
Interactive-Model [13]	0.7515 (0.6631, 0.8324)	0.6710 (0.5812, 0.7564)	0.6222 (0.5012, 0.7382)	0.6909 (0.5842, 0.7931)	0.5231 (0.4214, 0.6215)	0.4588 (0.3531, 0.5594)	0.8185 (0.7423, 0.8872)
TMSS [48]	0.7010 (0.6123, 0.7842)	0.6710 (0.5814, 0.7562)	0.3556 (0.2124, 0.4952)	0.8000 (0.7012, 0.8941)	0.3541 (0.2312, 0.4721)	0.5382 (0.3842, 0.6852)	0.7546 (0.6712, 0.8342)
Integrated-Model [49]	0.7626 (0.6742, 0.8461)	0.6581 (0.5742, 0.7362)	0.5556 (0.4321, 0.6742)	0.7000 (0.5912, 0.8042)	0.4816 (0.3812, 0.5794)	0.4586 (0.3524, 0.5614)	0.7992 (0.7214, 0.8712)
MRI-RNA [24]	0.7586 (0.6632, 0.8514)	0.7871 (0.7123, 0.8582)	0.6000 (0.4741, 0.7214)	0.8636 (0.7688, 0.9573)	0.6144 (0.5012, 0.7231)	0.6491 (0.5312, 0.7624)	0.8431 (0.7714, 0.9124)
CITR-Net [25]	0.7788 (0.6912, 0.8624)	0.6968 (0.6124, 0.7782)	0.4444 (0.3214, 0.5632)	0.8000 (0.7042, 0.8912)	0.4311 (0.3214, 0.5384)	0.4742 (0.3612, 0.5842)	0.7882 (0.7124, 0.8612)
AER-SwinT [32]	0.7434 (0.6532, 0.8312)	0.6710 (0.5824, 0.7542)	0.5111 (0.3921, 0.6254)	0.7364 (0.6412, 0.8272)	0.4591 (0.3512, 0.5624)	0.4484 (0.3342, 0.5582)	0.7947 (0.7231, 0.8624)
**BPMINet (ours) **	**0.8475** (**0.7356, 0.9594**)	**0.8452** (**0.7366, 0.9538**)	**0.7778** (**0.5470, 0.9989**)	**0.8727** (**0.7756, 0.9698**)	**0.7406** (**0.5503, 0.9309**)	**0.7190** (**0.5469, 0.8911**)	**0.9088** (**0.8220, 0.9956**)

Note: Bold values indicate the best performance among all compared methods.

**Table A2:** Evaluation results of our BPMINet model and all other comparison models on the MAMA-MIA dataset. For all metrics, 95% confidence intervals (CIs) are provided in the second line of each cell.

Methods	AUC (95% CI)	ACC (95% CI)	SEN (95% CI)	SPE (95% CI)	F1 (95% CI)	PPV (95% CI)	NPV (95% CI)
BERT [43]	0.6503 (0.5986, 0.7022)	0.6321 (0.5816, 0.6826)	0.5054 (0.4274, 0.5832)	0.6893 (0.6324, 0.7388)	0.4608 (0.3815, 0.5406)	0.4234 (0.3461, 0.5012)	0.7553 (0.6854, 0.8258)
MLP [44]	0.6513 (0.5823, 0.7218)	0.6756 (0.6187, 0.7291)	0.3871 (0.2911, 0.4853)	0.8058 (0.7375, 0.8655)	0.4260 (0.3484, 0.5073)	0.4737 (0.3500, 0.5938)	0.7444 (0.6794, 0.8049)
DenseNet [45]	0.5432 (0.4722, 0.6140)	0.6187 (0.5487, 0.6871)	0.2796 (0.1932, 0.3647)	0.7718 (0.7150, 0.8284)	0.3133 (0.2293, 0.3981)	0.3562 (0.2641, 0.4607)	0.7035 (0.6473, 0.7534)
ConVit [46]	0.5451 (0.4748, 0.6163)	0.6321 (0.5821, 0.6853)	0.4194 (0.3401, 0.5035)	0.7282 (0.6649, 0.7872)	0.4149 (0.3478, 0.4865)	0.4105 (0.3377, 0.4901)	0.7353 (0.6683, 0.8021)
ResNet-50 [47]	0.5359 (0.4521, 0.6197)	0.6087 (0.5452, 0.6722)	0.2043 (0.1241, 0.2847)	0.7913 (0.7261, 0.8568)	0.2452 (0.1821, 0.3089)	0.3065 (0.1379, 0.4828)	0.6878 (0.6290, 0.7410)
ViT [34]	0.6053 (0.5392, 0.6724)	0.6455 (0.5591, 0.7322)	0.4086 (0.3027, 0.5143)	0.7524 (0.6632, 0.8452)	0.4176 (0.3358, 0.4998)	0.4270 (0.2500, 0.6043)	0.7381 (0.6625, 0.8132)
SIDLN [30]	0.6688 (0.6050, 0.7295)	0.6421 (0.5741, 0.7114)	0.4946 (0.4251, 0.5622)	0.7087 (0.6444, 0.7650)	0.4623 (0.3864, 0.5385)	0.4340 (0.3711, 0.4993)	0.7565 (0.6604, 0.8431)
BERT-ViT	0.5854 (0.5036, 0.6682)	0.6589 (0.5842, 0.7128)	0.3763 (0.2764, 0.4731)	0.7864 (0.7383, 0.8351)	0.4070 (0.2538, 0.5617)	0.4430 (0.1975, 0.6875)	0.7364 (0.6712, 0.8014)
Interactive-Model [13]	0.5423 (0.4849, 0.6020)	0.6555 (0.5875, 0.7215)	0.2903 (0.2086, 0.3741)	0.8204 (0.7667, 0.8744)	0.3439 (0.2877, 0.4089)	0.4219 (0.3498, 0.4885)	0.7191 (0.6364, 0.7908)
TMSS [48]	0.6170 (0.5406, 0.6845)	0.6187 (0.5617, 0.6724)	0.4624 (0.3636, 0.5647)	0.6893 (0.6321, 0.7391)	0.4300 (0.3632, 0.5002)	0.4019 (0.3234, 0.4787)	0.7396 (0.6776, 0.8000)
Integrated-Model [49]	0.6938 (0.6423, 0.7456)	0.6823 (0.6165, 0.7469)	0.3763 (0.3101, 0.4470)	0.8204 (0.7750, 0.8653)	0.4242 (0.3529, 0.5061)	0.4861 (0.3279, 0.6445)	0.7445 (0.6880, 0.8015)
MRI-RNA [24]	0.6905 (0.6222, 0.7484)	0.6923 (0.6418, 0.7461)	0.4516 (0.3727, 0.5326)	0.8010 (0.7482, 0.8530)	0.4773 (0.3989, 0.5568)	0.5060 (0.3953, 0.6203)	0.7639 (0.7040, 0.8165)
CITR-Net [25]	0.6946 (0.6274, 0.7533)	0.7124 (0.6573, 0.7638)	0.4624 (0.3516, 0.5652)	0.8252 (0.7691, 0.8798)	0.5000 (0.4196, 0.5794)	0.5443 (0.4742, 0.6151)	0.7727 (0.7072, 0.8306)
AER-SwinT [32]	0.7008 (0.6329, 0.7637)	0.7023 (0.6455, 0.7525)	0.4946 (0.3928, 0.5976)	0.7961 (0.7418, 0.8498)	0.5083 (0.4099, 0.5923)	0.5227 (0.4167, 0.6292)	0.7773 (0.7158, 0.8291)
**BPMINet (ours) **	**0.7370** (**0.6730, 0.7953**)	**0.7391** (**0.6854, 0.7862**)	**0.5376** (**0.4338, 0.6324**)	**0.8301** (**0.7812, 0.8814**)	**0.5618** (**0.4687, 0.6428**)	**0.5882** (**0.4820, 0.6956**)	**0.7991** (**0.7401, 0.8487**)

Note: Bold values indicate the best performance among all compared methods.
